# The Evolution of Vaccines Development across *Salmonella* Serovars among Animal Hosts: A Systematic Review

**DOI:** 10.3390/vaccines12091067

**Published:** 2024-09-18

**Authors:** Abubakar Siddique, Zining Wang, Haiyang Zhou, Linlin Huang, Chenghao Jia, Baikui Wang, Abdelaziz Ed-Dra, Lin Teng, Yan Li, Min Yue

**Affiliations:** 1 Key Laboratory of Systems Health Science of Zhejiang Province, School of Life Sciences, Hangzhou Institute for Advanced Study, University of Chinese Academy of Sciences, Hangzhou 310024, China; abubakars@zju.edu.cn (A.S.); wangbaikui@zju.edu.cn (B.W.); tenglinchn@zju.edu.cn (L.T.); 2 Department of Veterinary Medicine, College of Animal Sciences, Zhejiang University, Hangzhou 310058, China; znwang@zju.edu.cn (Z.W.); zhouhaiyang@zju.edu.cn (H.Z.); 22217045@zju.edu.cn (L.H.); 22017118@zju.edu.cn (C.J.); yanli3@zju.edu.cn (Y.L.); 3 Hainan Institute of Zhejiang University, Sanya 572025, China; 4 Laboratory of Engineering and Applied Technologies, Higher School of Technology, M’ghila Campus, BP: 591, Beni Mellal 23000, Morocco; abdelaziz_iaa@yahoo.fr; 5 State Key Laboratory for Diagnosis and Treatment of Infectious Diseases, National Clinical Research Center for Infectious Diseases, National Medical Center for Infectious Diseases, The First Affiliated Hospital, College of Medicine, Zhejiang University, Hangzhou 310003, China

**Keywords:** bacterial vaccines, *Salmonellosis*, conventional vaccine technologies, reverse vaccinology, immunotherapy, infectious diseases

## Abstract

*Salmonella* is a significant zoonotic foodborne pathogen, and the global spread of multidrug-resistant (MDR) strains poses substantial challenges, necessitating alternatives to antibiotics. Among these alternatives, vaccines protect the community against infectious diseases effectively. This review aims to summarize the efficacy of developed *Salmonella* vaccines evaluated in various animal hosts and highlight key transitions for future vaccine studies. A total of 3221 studies retrieved from Web of Science, Google Scholar, and PubMed/Medline databases between 1970 and 2023 were evaluated. One hundred twenty-seven qualified studies discussed the vaccine efficacy against typhoidal and nontyphoidal serovars, including live-attenuated vaccines, killed inactivated vaccines, outer membrane vesicles, outer membrane complexes, conjugate vaccines, subunit vaccines, and the reverse vaccinology approach in different animal hosts. The most efficacious vaccine antigen candidate found was recombinant heat shock protein (rHsp60) with an incomplete Freund’s adjuvant evaluated in a murine model. Overall, bacterial ghost vaccine candidates demonstrated the highest efficacy at 91.25% (95% CI = 83.69–96.67), followed by the reverse vaccinology approach at 83.46% (95% CI = 68.21–94.1) across animal hosts. More than 70% of vaccine studies showed significant production of immune responses, including humoral and cellular, against *Salmonella* infection. Collectively, the use of innovative methods rather than traditional approaches for the development of new effective vaccines is crucial and warrants in-depth studies.

## 1. Introduction

Salmonellosis, caused by a wide range of *Salmonella* serovars, is one of the leading bacterial diseases in both humans and animals [1]. It is reported that *Salmonella* mainly consists of two species: *Salmonella bongori* and *Salmonella enterica*, with over 2600 serovars discovered so far [2]. These serovars can be grouped into typhoidal *Salmonella* (TS) and nontyphoidal *Salmonella* (NTS) based on their disease syndromes and host ranges [3,4]. Typhoidal *Salmonella* (TS) serovars are restricted to one host species, whereas NTS serovars have diverse hosts, including humans and animals with mild to moderate gastrointestinal syndromes [5]. *Salmonella* annually causes an estimated 1.35 million infections, 26,500 hospitalizations, and 420 deaths in the United States alone, leading to an estimated economic burden of over $3.7 billion. Globally, there are approximately 93 million NTS infections, and 155,000 deaths occur annually [6].

Salmonellosis is considered as the third leading cause of mortality among food-borne illnesses [7]. The majority of human salmonellosis cases are food-borne, mainly directly or indirectly linked to animal or human fecal contamination [8]. Infections can also spread through direct or indirect contact with animals and animal-associated food products [9,10,11]. In recent years, sub-Saharan Africa and southern Asia have witnessed a persistent rise in invasive NTS infections in both adults and children [12]. Notably, *S.* Typhimurium and Enteritidis were responsible for over 80% of invasive nontyphoidal *Salmonella* (iNTS) cases [13]. Therefore, the control of these specific *Salmonella* serovars at the animal interface is essential for preventing transmission of infections to humans. Antibiotics are commonly used to treat bacterial infections in humans as well as animals. Although the therapeutic use of antimicrobials has revolutionized modern medicine, the emergence of multidrug-resistant (MDR) bacterial strains has led to a significant antimicrobial resistance (AMR) crisis [10]. The high mortality and morbidity caused by MDR TS and NTS highlighted the urgent need for alternate therapies against *Salmonella* [14] dissemination and infection, such as vaccines, probiotics, and prebiotics.

Vaccines stimulate an immunological response in the host to combat infection. The field of vaccinology has produced several effective vaccines that have substantially reduced the burden of deadly pathogens, i.e., *Salmonella* in animals and humans [15]. Historically, vaccines have been produced using different methods, including live attenuated (weakened) or inactivated (killed). However, both strategies have their shortcomings. Conventional vaccinations (attenuated or killed) are typically costly to produce, require adjuvants (inactivated vaccines) and multiple doses (live attenuated and inactivated vaccine) to induce adequate immunity, can interfere with maternal antibodies (live attenuated, inactivated), and offer little or no protection. Considering all of these challenges, continuous research is needed for the development of effective and safe vaccines [16]. Therefore, conventional vaccines have undergone considerable improvements, including mutant-attenuated (live-attenuated) and subunit vaccines (a type of inactivated vaccine containing part of the bacteria or virus) over the years. Despite these advancements, only a few licensed commercial *Salmonella* vaccines are available (Table 1), which include live-attenuated vaccines, killed inactivated vaccines, and a few subunit vaccines [17]. Recently, the introduction of new biotechnological approaches in vaccine development generation has led to the development of potential next-generation vaccines, such as recombinant subunit vaccines, DNA vaccines, mRNA vaccines, bacterial ghost (BGs) vaccines, and reverse vaccinology [18]. DNA, bacterial ghost, and mRNA vaccines, when produced through recent developments in molecular biological techniques, induce robust immune responses against pathogens [19]. Another innovative approach to vaccine development is reverse vaccinology, which combines genomics, proteomics, and bioinformatics to identify new genes in pathogens that could elicit immune response [20]. However, the development of vaccines remains challenging due to the various *Salmonella* serovars and their unique pathogenic mechanisms.

Therefore, this study provides a comprehensive overview of the developed vaccines for TS and NTS serovars in different animal hosts. Additionally, we used a systematic review approach to gather pertinent studies regarding the safety and efficacy of developed conventional and next-generation vaccines assessed in various animal hosts for this review, which is primarily intended for a broad scientific audience. This review will provide insights into the key issues that veterinary immunologists are currently facing.

## 2. Methods

### 2.1. Systematic Literature Search

A systematic review followed the Preferred Reporting Items for Systematic Reviews and Meta-Analyses (PRISMA) guidelines [21] to address key research questions. The key question focused on identifying potential vaccine antigens against diverse *Salmonella* serovars and determining the most effective, influential vaccine candidates. Three electronic databases, including Elsevier ScienceDirect, Scopus, and PubMed Central, were searched to identify relevant studies for this review, using the following terms: “Salmonella”, “Vaccine”, and “Animal Models”. The search was conducted in December 2023, and only English-language research articles published until 30 November 2023, were considered.

### 2.2. Selection Criteria

A two-step procedure consisting of primary and secondary inclusion/exclusion criteria was used to determine the eligibility of studies for inclusion in this review (Table 2). The review excluded evaluating vaccine effects in clinical settings due to variations in immune responses and colonization between animal models and human trials [22]. In cases where multiple sample types (i.e., ceca, cloaca, liver, and spleen) were assessed within a single trial, the cecal sample result was chosen for this study [9]. When necessary, if information on vaccine efficacy data was unavailable, the author (AS) emailed the corresponding author of the article to obtain the missing information.

Vaccine efficacy and study eligibility for this review were assessed based on reductions in *Salmonella* load in the intestine and other organs during postmortem examination, as commonly used methods to evaluate the effectiveness of different *Salmonella* control strategies in various animal models [22]. Host-adapted serovars are all evaluated in the respective animal hosts, whereas *S*. Typhi serovar is restricted to humans; humanized mice are used as model animals for preclinical studies. Consequently, studies reported vaccination efficacy by analyzing the prevalence or proportion of “diseased” (i.e., colonized) or decreased levels of *Salmonella* colonization in both vaccinated and unvaccinated groups after the *Salmonella* challenge.

### 2.3. Data Extraction and Analysis

All research publications relevant to *Salmonella* vaccine studies were imported into Microsoft Excel datasheets, where duplicate studies were manually removed. Initially, one author (AS) studied the article to determine whether they fulfilled the inclusion criteria. If the titles and abstracts matched the selection criteria, the complete text of each potential publication was examined for the final evaluation of eligible studies. The complete text was examined during this stage to classify the qualifying studies based on the vaccine-efficacy studies and extract relevant information. The final lists of eligible articles were entered into the EndnoteX9 application for storage and consolidation.

The extracted information of the eligible studies comprised article identification, information about animal models, vaccine candidates, *Salmonella* challenge serovars, and vaccine efficacy, safety, and immune responses among vaccinated subjects. In cases where multiple trials were conducted in a single study, we only evaluated the trials involving immune responses and proliferation assays. Vaccine candidates prepared using reverse vaccinology but not assessed in an in vivo model were excluded. Only three vaccine studies used the reverse vaccinology approach: two in the murine model and one in the chicken type. The extracted information was summarized in Microsoft Excel datasheets (Supplementary Table S1).

### 2.4. Data Analysis

The statistical analysis was performed using the GraphPad Prism v10.1.2 software. The pooled Efficacy of vaccines was calculated using the random-effects model with a 95% confidence interval (95% CI) with Metaprop order [23]. Descriptive statistics were imported into Microsoft Excel for graphic analysis. Statistical significance was determined at *p*-values < 0.05.

## 3. Results

### 3.1. An Assembly of Quantified Studies

**Figure 1:**
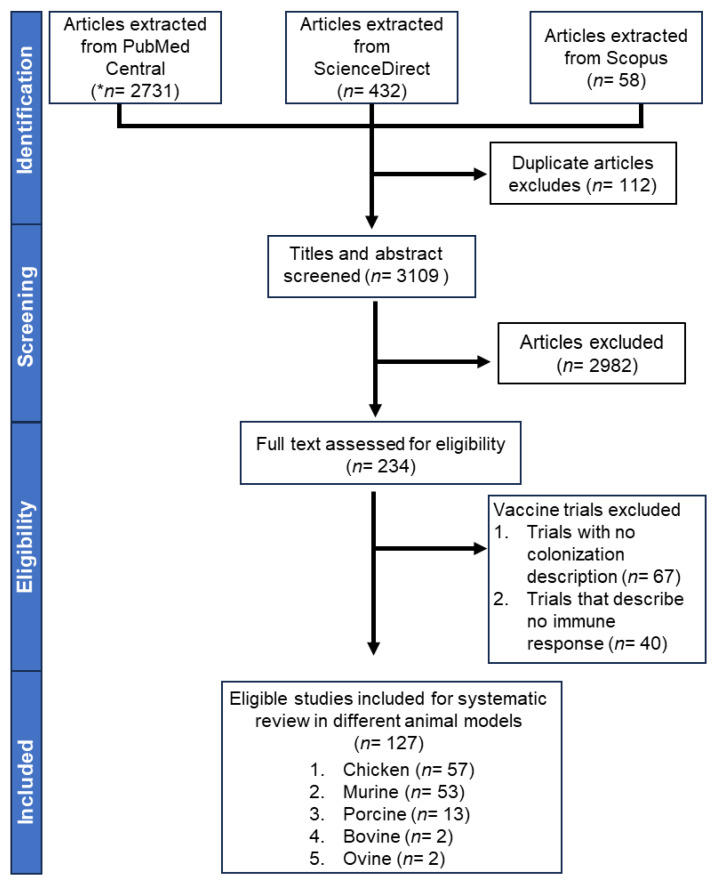
A simplified PRISMA diagram of methodology. A total of 3221 articles from different animal models were identified by our search strategy from different online databases (i.e., PubMed, ScienceDirect, and Google Scholar). * *n* is the no. of studies.

During the initial search, a total of 3221 publications were identified from three databases. After removing duplicates, the remaining 3109 articles (96.5%) were evaluated based on their abstracts. After reviewing the titles and abstracts, 2982 publications were eliminated (Figure 1). Among the excluded articles, 857 were review articles. Additionally, there were 372 articles related to control and pathogenesis of *Salmonella*. Moreover, 105 articles were related to clinical studies and knowledge, attitudes, and practice (KAPE) studies, 98 were related to other alternatives to antibiotics (probiotics, prebiotics, and natural products), and 75 were related to bacteriophages and parasites. Furthermore, 49 articles were related to bacteria other than *Salmonella*, 47 were related to only antigen preparation, 45 were related to One Health, and 41 were related to food safety issues. Additionally, there were 36 articles related to guidelines for the prevention and diagnosis of infections, 13 articles related to only methods for antigen preparation, and three articles lacking full text. A total of 1244 primary research studies did not meet the inclusion criteria: 466 were non-*Salmonella* studies, 421 were *Salmonella* studies but not related to vaccines, 245 were reverse-vaccinology vaccine candidate research without animal models, and 112 were *Salmonella* vaccines conducted in clinical trials, immunogenicity experiments, or articles with full-text unavailability. In the end, a total of 127 qualified studies that met the criteria were included in the systematic review.

### 3.2. Animal Models

**Figure 2:**
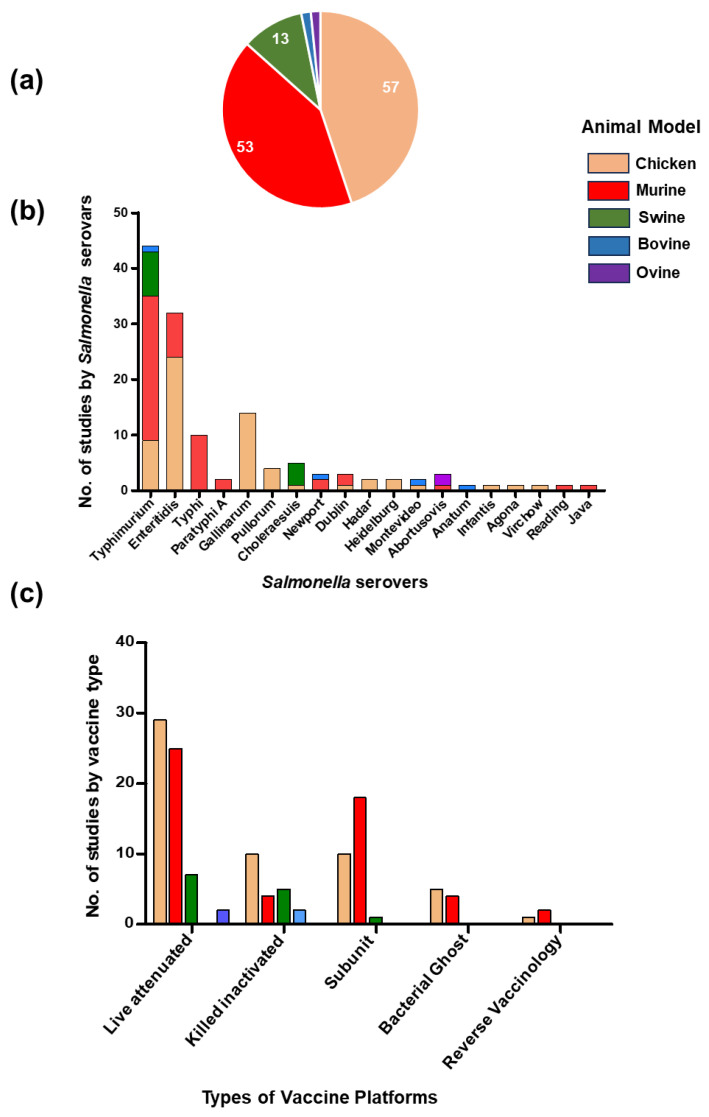
Summary of vaccine studies used in this review. (**a**) Proportion of vaccine studies according to animal models; (**b**) types of different *Salmonella* serovars used in different vaccine studies; (**c**) types of vaccine strategies used in this study.

In this systematic review, five animal hosts (chicken, swine, bovine, ovine, and murine) were used to evaluate the efficacy and protective properties of conventional and reverse vaccinology approach vaccines in preventing *Salmonella* infections (Figure 2a). A total of 57 vaccine studies (59 trials) were conducted in the chicken model, 53 studies (55 trials) in the murine model, 13 studies (20 trials) in swine, two studies in bovine, and two studies in ovine. During our search, we identified 19 different serovars of *Salmonella*, encompassing both typhoidal and non-typhoidal, targeted by a vaccine. Notably, *S*. Typhimurium emerged as one of the most extensively researched serovars, serving as a challenge infection during vaccination trials across all animal models, followed by *S*. Enteritidis (Figure 2b).

### 3.3. Vaccine Types

Overall, eight types of vaccine strategies (live-recombinant, mutant-attenuated, subunit, a combination of vaccines, killed whole-cell, cell lysate, crude-cell lysate, and bacterial ghost vaccines) and two types of reverse vaccinology approach vaccines (single-peptide, multi-epitope) (Table 3) were classified.

Based on the investigation of 127 studies analyzing *Salmonella* loads in the ceca, liver, or spleen of the vaccinated and non-vaccinated mice, chicken, swine, ovine, and bovine, live-attenuated vaccine candidates were the most frequently used (61 studies), followed by subunit vaccines (24 studies) and inactivated killed vaccine (12 studies) (Figure 2c). The majority of investigations in swine focused on the development of live-attenuated vaccination candidates (*n* = 12), highlighting the importance of assessing the efficacy of new-generation vaccinations in combating *Salmonella* infection within the swine industry. Bacterial ghost vaccines were also used in mice and chicken models to combat multidrug-resistant *Salmonella* serovars. Only three trials (one in the chicken model and two in the mouse model) were found in which antigens were prepared using a reverse vaccinology approach.

**Figure 3:**
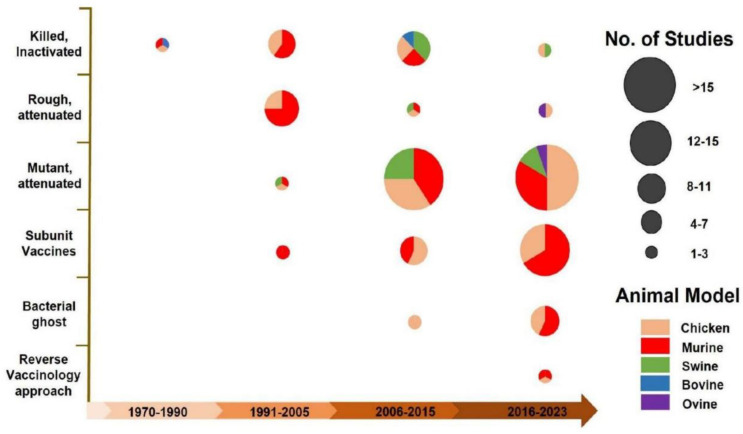
Transition in the development of different vaccine candidates across various animal models from 1970 to 2023.

The shift in vaccination types in different animal models between 1970 and 2023 is shown (Figure 3). Initially, killed inactivated, rough attenuated, and few mutant-attenuated strains (*galE* mutant strain of *S*. Typhi, *aroA* and *cya crp* double-deletion strategy, and *galE* mutant strain of *S*. Gallinarum) vaccines were evaluated in mice, bovine, swine, and chickens before the year 2000. However, over the past decade, there has been a noticeable increase in the use of mutant-attenuated, subunit, and bacterial ghost vaccines in different animal models. Vaccine candidates prepared through the reverse vaccinology approach have also been evaluated in the chicken and murine models recently.

### 3.4. Vaccine Antigens

This review identified 13 antigens from selected vaccine studies using criteria related to vaccine efficacy (relative reduction of disease risk and mortality) (Supplementary Table S1). Live attenuated vaccine candidates were the most frequently assessed antigen in different animal hosts, including chickens (*n* = 22), mice (*n* = 25), swine (*n* = 10), and bovine (*n* = 2). Live-attenuated vaccine candidates (roughly attenuated or mutant-attenuated) were administered via oral, subcutaneous, or intramuscular (IM) routes as a single dose or booster. Metabolic mutations (Δ*asd*, Δ*rpoS*, and Δ*phoP*) or amino acid (Δ*aroA*) were used for attenuation in different studies (*n* = 14). Flagellin proteins (*fliA*, *fliB*, *fliC*), used in eight trials (eight papers), were evaluated in two different vaccine types (mutant-attenuated and subunit vaccines) and different routes of administration (orally, intramuscularly, or subcutaneously with a booster). Formalin- and acetone-inactivated vaccines were used in seven trials in three animal models, including chickens (*n* = 3), mice (*n* = 2), and swine (*n* = 2) orally and subcutaneously.

Eleven studies were conducted using three antigens, including capsular polysaccharide, entire cell lysate, and crude cell lysate, to assess their effectiveness in homologous challenges across several animal models. The efficacy of core and O-polysaccharide (COPS) vaccine antigens, both with and without adjuvant, was assessed using subunit vaccination methods (oral, subcutaneous, and intramuscular with booster) and COPS vectored vaccines (oral with booster) after challenge with different *Salmonella* serovars. The antigenicity of both the total outer membrane proteins (OMP) and vesicles employed in the crude lysate vaccine was assessed in only two animal models, namely mice (*n* = 8) and chickens (*n* = 7), after a different *Salmonella* serovars challenge. The antigen was administered either as biodegradable and biocompatible nanoparticles (OMP-NP) or directly without encapsulation through oral or subcutaneous vaccinations, followed by a booster. Bacterial ghost vaccines (empty bacterial envelopes) using mutant-attenuated strains as antigens were tested in nine studies in two animal models. Chickens (*n* = 5) and mice (*n* = 4) were administered orally, intramuscularly, and subcutaneously.

Reverse vaccinology is an innovative method that combines immunology, computational biology, structural biology, and microbial genetics to identify and design vaccine antigens. Several studies have been conducted in which vaccine candidates were prepared and analyzed using bioinformatic tools, but they were not tested in animal models. Here, only three articles were identified in which vaccine antigens were identified and prepared using a reverse vaccinology approach and then tested in animal models; one study was conducted in chickens and two in mice models.

### 3.5. Vaccine Efficacy Evaluated by Organ Bacterial Colonization

This analysis found three different outcomes regarding the efficacy of vaccination, as reported in the 127 studies: no reduction in bacterial load in organs (intestine and liver) (*n* = 9), non-significant log10 bacterial load reductions (*n* = 27), and significant log10 bacterial load reductions (*n* = 96) (Supplementary Table S1).

Among 96 studies in which significant log10 bacterial load reductions occurred, 84 vaccine studies in different hosts, including mice (*n* = 39), chickens (*n* = 33), swine (*n* = 10), bovine (*n* = 1), and ovine (*n* = 1), exhibited significant log_10_ reductions ranging between 1.0-log_10_ and 4.0-log_10_ of *Salmonella* serovars loads in the intestine and liver after challenge with different *Salmonella* serovars. A subunit vaccine candidate, recombinant heat shock protein (rHsp60), derived from gram-negative bacteria with incomplete Freund’s adjuvant antigen, caused the most significant decrease in bacterial load in the intestine, with a reduction of 4.0-log_10_ after *S*. Enteritidis infection in mice model [37]. Furthermore, bacterial ghost cells carrying a heat-labile enterotoxin B component from *S*. Typhimurium result in a 3.7-log10 reduction in bacterial load in the caecum of chicken after *S*. Typhimurium challenges, compared to non-vaccinated animals. While all the other studies (*n* = 12) reported significant reductions (≤1 log_10_) in *Salmonella* loads after challenging. Moreover, there was just one study conducted on sheep to assess the effectiveness of the vaccine against *S*. Abortusovis, with results showing a 2.0-log_10_ reduction in feces after challenge infection. All three antigens identified through the reverse vaccinology approach significantly reduced the *Salmonella* load in both mouse and chicken models.

Twenty-seven *Salmonella* vaccine trials performed in swine, mice, sheep, and bovine showed a non-significant reduction upon challenge. These trials included live mutant or rough inactivated vaccines given orally with or without a booster, crude lysate vaccines with outer membrane protein (OMP), OMP-NP given orally with the booster, outer membrane vesicles (OMVs) given orally and subcutaneously, and formalin-killed with mineral oil adjuvant given orally and intramuscularly with and without a booster.

Nine trials from mice (*n* = 6) and chickens (*n* = 3) used rough and mutant-attenuated with and without a booster, outer membrane vesicles (OMVs), COPS, and the flagellar monomer protein (*fliC*) conjugate, and a recombinant *fliC* protein failed to reduce *Salmonella* load in organs.

### 3.6. Vaccine Safety, Efficacy, and Immune Responses

**Figure 4:**
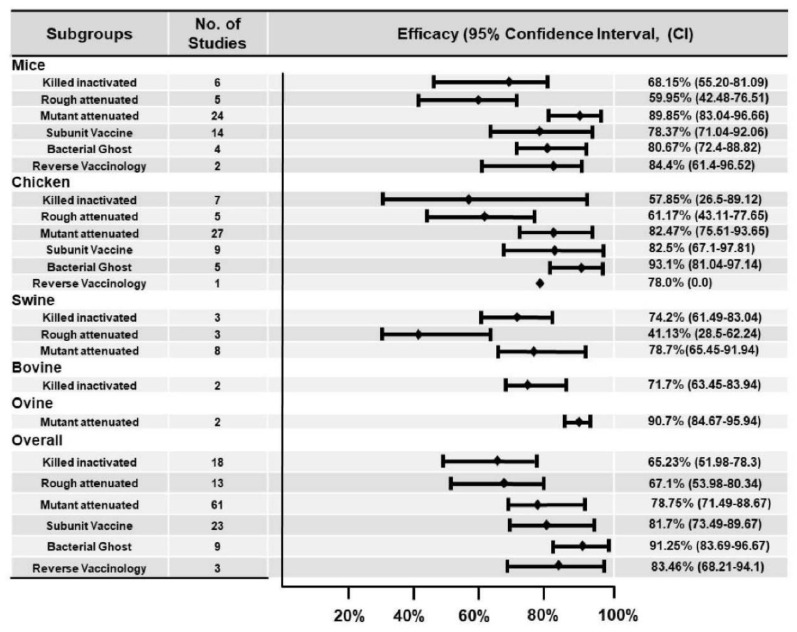
Efficacy of different vaccine candidates in various animal models. Here, the small square represents the reported efficacy after vaccination. All data were taken from the articles included in this systematic review. The 95% confidence intervals were not evenly distributed because the response levels for all vaccines were not the same. There is only one reverse vaccinology study in the chicken model, so we were not able to find the upper and lower limits of 95% CI; thus, it was not reported in the figure.

This study analyzed high-quality studies to evaluate vaccine efficacy by comparing mortality rates between vaccinated and unvaccinated groups. Additionally, immunological responses and vaccination safety were assessed. Various levels of vaccination efficacy were observed in vaccinated animals, ranging from 100% inhibition of *Salmonella* serovar colonization to no effect. The effectiveness of each vaccine candidate is presented (Supplementary Table S2). According to our results, out of 130 trials conducted in various studies, the majority (36 trials) were in mice, whereas 30 trials in chickens and 10 trials in swine used live recombinant mutant antigens and showed vaccine efficacy of >75%. Collectively, we observed efficacy of 89.85% (95% CI = 83.04–96.66) in mice, 82.47% (95% CI = 75.51–93.65) in chickens, and 78.7% (95% CI = 65.45–91.94) in swine for mutant-attenuated antigens (Figure 4). Only four investigations showed no adequate protection after vaccination: one from mice, using rough attenuated vaccines; two from chicken using killed inactivated vaccines; and one from swine, using rough attenuated vaccines. In the swine model, rough attenuated antigens had the lowest efficiency of 41.13% (95% CI = 28.5–62.24), followed by 57.85% (95% CI = 26.5–89.12) in the chicken model for killed inactivated antigens. Antigens generated using the reverse vaccinology approach were also utilized to examine vaccine efficacy, but two investigations on mice revealed vaccine efficacy of less than 70%, and one on poultry revealed vaccination efficacy of 75%. According to our study findings, the bacterial ghost vaccine strategy produced the most efficient vaccine candidates, 91.25% (95% CI = 83.69–96.67), followed by the reverse vaccinology approach (83.46% (95% CI = 68.21–94.10) and subunit vaccines (81.7% (95% CI = 73.49–89.67). The live mutant attenuated vaccine candidates also showed good efficacy (78.75%; 95% CI = 71.49–88.67) in different animal models.

The immunological responses produced by vaccines played a crucial role in conferring protection against salmonellosis in various hosts. This systematic review article included 127 published studies on the immunogenicity of *Salmonella* vaccines that met the inclusion criteria. Overall, these trials found that more than 70% (n = 95) of vaccine studies showed significant production of antibodies against *Salmonella* infection. Only seven studies (rough attenuated = three, mutant attenuated = two, dead inactivated = two) (5.5%) found no effect on immune response against *Salmonella* infection. The ability of the *Salmonella* vaccines to induce cell-mediated immunity among different animal hosts was also assessed (Supplementary Table S2)

Vaccine safety is another important criterion for evaluating vaccine efficacy. Local effects, including pain and swelling at the vaccination site, and systemic effects, such as fever, fatigue, and diarrhea (mild and moderate adverse effects), were reported as the side effects of *Salmonella* vaccination. The majority of *Salmonella* vaccine antigens produced no symptoms or adverse effects in animals after vaccination. However, few vaccine trials conducted in mice, chickens, swine, and bovine using rough and mutant-attenuated strains revealed mild-to-moderate adverse effects on the hosts. Nevertheless, none of the antigens derived from reverse vaccinology exhibited adverse effects on the animal hosts.

## 4. Discussion

The emergence of MDR *Salmonella* serovars from animals significantly threatens public health. Effective vaccines are often used as an alternative to antibiotics to minimize the risk of *Salmonella* infections in different hosts [53]. This systematic literature review aimed to assess the efficacy of high-qualified *Salmonella* vaccine studies conducted in various animals and highlight the transition for future vaccine developments with the advancement of biotechnological approaches. The efficacy of *Salmonella* control measures is often assessed by measuring the decrease in the prevalence (in percentage) of *Salmonella* load in the intestine or other tissues after challenge [54,55]. Furthermore, the efficacy of vaccines is evaluated by assessing the immunological response and safety profile of vaccines tested in different animal hosts [56].

In this review, the majority of the studies indicate that vaccination leads to enhanced immune responses to different types of vaccine antigens and reduced bacterial burdens in the intestines and other organs in different animal hosts [35,38,44,52,57,58,59,60,61,62,63,64,65,66]. Numerous studies indicate significant decreases in *Salmonella* loads in mice, chickens, and swine; however, the extent of these reductions varies considerably. Divergence in vaccine response among various animal hosts can be attributed to their genetic background, physiological state, and immunological responses. Large animal species like pigs, cows, and sheep are physiologically and immunologically closer to humans and often are host to the same or closely related infections [67]. However, it is challenging to assess the possible effects of these investigations on the risk of *Salmonella* transmission to humans.

In total, 127 studies met the criteria for selection and were included. These trials evaluated different *Salmonella* serovars, including TS and NTS loads, in vaccinated mice [68,69,70,71], chickens [72,73], pigs [25,74,75], bovine [17,76], and sheep [4]. The reason for including a murine model here is that most therapeutic vaccines rely on sophisticated and extensive studies in mice [40], whereas other animals are employed as a food supply, and it is directly linked to *Salmonella* transmission to humans [77,78]. The present review focuses on the development of vaccine candidates from various *Salmonella* serovars through different approaches. For example, serovars Typhi and Paratyphi A/B/C are mainly adapted to the human host, Choleraesuis is mainly limited to swine, Abortusovis is mainly limited to goats and sheep, and Dublin is mainly limited to cows, while Gallinarum and Pullorum are restricted to poultry. These serovars are then employed to induce infection in different animals, allowing for the assessment of vaccination effectiveness. Since *S*. Typhi serovar is restricted to humans, humanized mice are used as model animals to study *S*. Typhi. *S*. Typhimurium was one of the most researched serovars as a challenge infection during vaccination trials in all animals, followed by *S*. Enteritidis and *S*. Typhi. According to previous reports, *S*. Typhimurium is one of the most prevalent serovars detected in animals and food, and it is a serovar well-adapted for transfer to humans, where it causes pathogenesis [10,71]. According to reports from sub-Saharan Africa, *S.* Typhimurium can cause invasive infections in humans known as invasive nontyphoidal salmonellae [iNTS] that are comparable to typhoid forms [79]. *Salmonella* serovars are commonly found in the gastrointestinal tracts of animals and can be transmitted to humans through feces and other organs [80]. As a result, MDR *Salmonella* must be controlled at the animal interface in order to prevent transmission to humans.

The differences in bacterial loads (log_10_) in the ceca, liver, or spleen between vaccinated and non-vaccinated animals were used to determine vaccine efficacy in these trials [71,81,82]. In this study, we found that numerous vaccine candidates demonstrated significant reductions in *Salmonella* loads in vaccinated animals’ intestines and other organs, ranging from 1.0 to 4.0-log10 reductions, when compared to non-vaccinated animals, as reported in various trials [72,83,84]. However, the efficacy of the vaccination was found to have no significant effect on the reduction of *Salmonella* colonization in a few studies [70,85,86], suggesting that the statistical data was insufficient to distinguish between vaccinated and non-vaccinated groups. Therefore, more clinical investigations are needed to identify the appropriate vaccine trial parameters in order to precisely determine reductions and their impact on the risk of human transmission. Defining these characteristics is critical for measuring the efficacy of *Salmonella* vaccines, which are likely to remain dependent on challenge tests. Several studies have revealed weak connections between immune responses and a decrease in the bacterial burdens in the intestines and other organs of animals during challenge studies [25,42,43].

This study includes only three investigations in which potential antigens, identified through a reverse vaccinology approach, were tested in mice [87,88] and chicken [52], resulting in a significant reduction (*p* ≤ 0.05) in *Salmonella* load. However, most of the potential vaccine candidates identified through the reverse vaccinology approach were only validated by in silico analysis [87,89,90]. Therefore, there is a need for these potential vaccine candidates to be tested in animal models for evaluation of vaccine efficacy and safety. Another aspect critical for evaluating vaccine efficacy involves assessing immune responses within the host. This study provides a comprehensive evaluation of the existing literature on *Salmonella* vaccines in various animal hosts. More than 70% (*n* = 95) of vaccine studies produced significant antibodies against *Salmonella* infection. The appearance of signs and symptoms, including inflammation, lethargy, and diarrhea observed after vaccination, provides insights into the vaccine’s safety profile [91]. A broad spectrum of safety profile data was identified, ranging from the absence of adverse effects (AEs) to the occurrence of mild and moderate AEs in animal models after vaccination. The vaccination program has been continued with the development of new vaccine candidates against *Salmonella* serovars. Our findings indicate that ongoing surveillance and randomized animal research on potential vaccine candidates against emerging MDR *Salmonella* serovars are critical.

Currently, no commercially available *Salmonella* vaccine with antigens produced through a reverse vaccinology approach exists. Current options are limited to a few vaccine candidates, including live-attenuated, killed-inactivated, and subunit vaccines, which are licensed and commercially available. Vaccination is a complex procedure that necessitates an in-depth knowledge of the host’s genetic components, cellular defenses, and interconnections. Therefore, continuous research is ongoing for developing potential vaccine candidates through novel approaches against infectious diseases. However, new research has revealed that various vaccine candidates developed through reverse vaccinology, mutant-attenuated vaccination, subunit vaccination, and bacterial ghost vaccines, identified as potential antigens (with efficacy exceeding 80%), could pave the way for commercial vaccine preparation. Despite the potential efficacy of these vaccination strategies, further study on immunological pathways is necessary to develop a vaccine that may effectively achieve the intended outcome while avoiding serious adverse effects like chronic stress. Researchers should focus more on developing vaccines with long-lasting immune responses. Over the past decade, notable advancements in vaccination have been achieved, expedited by the response to the COVID-19 epidemic [19]. Therefore, our approach may reveal new *Salmonella* vaccine candidates with improved efficacy and commercial viability.

The primary merit of the study is that it used strict bias-reduction strategies to interpret the most accurate results by reviewing high-quality publications published in reputable journals. This systematic review included only data from vaccine studies in which bacterial load, immune responses, and vaccine safety were estimated between vaccinated and non-vaccinated groups, as well as randomized trials in animal hosts, for a complete evaluation of the efficacy of the *Salmonella* vaccines. A limited number of trials or a single animal host were included in previous systematic review research on *Salmonella* vaccinations against different serovars in animal hosts. [47]. We incorporated a large number of investigations from various animal hosts against different *Salmonella* serovars in our study, indicating more precise data than previously demonstrated. [47,48]. The main drawback of this research is its inability to demonstrate the long-term protective effect of *Salmonella* vaccinations in animal hosts due to a lack of relevant literature.

## 5. Conclusions

This systematic analysis comprehensively integrated the most recent data on developed vaccine efficacy, immunogenicity, and safety in different animal hosts against *Salmonella* serovars. Over the last decade, immense progress has been made in establishing novel strategies to develop potential *Salmonella* vaccine candidates. In conclusion, mutant-attenuated, subunit, and bacterial ghost vaccines, as well as antigens prepared through reverse vaccinology, showed higher efficacy in different animal hosts. These next-generation vaccines are able to expedite the development timeline and can rapidly advance to commercialization in order to combat the spread of antibiotic-resistant *Salmonella*. Therefore, next-generation vaccines present a prospective route for improving the efficacy and safety of vaccine candidates, ultimately leading to better public health outcomes. This study also provides a comprehensive baseline dataset on the efficacy and safety of *Salmonella* vaccines against typhoidal and nontyphoidal serovars for future research.

## Figures and Tables

**Table 1:** A summary of commercial vaccines against salmonellosis in different animal models.

Vaccine Name	Company Name	Animal	General Information
POULVAC^®^	Zoetis, Parsippany, NJ, USA	Chicken	Mutant-attenuated *aroA*-deleted *Salmonella* Enteritidis and *S*. Typhimurium
SALMOVAC^®^	IDT Bio, Coralville, IA, USA	Chicken	Freeze-dried live-attenuated *S*. Enteritidis
NOBILIS 9R Vac^®^	CEVA Animal Health, Lenexa, KS, USA	Chicken	Live-attenuated *S*. Gallinarum
Fowlvax	Kenya Vaccine Institute, Nairobi, Kenya	Chicken	Live-attenuated *S*. Gallinarum
LAYERMUNE^®^	CEVA Animal Health, Lenexa, KS, USA	Chicken	Live-attenuated *S*. Enteritidis
CORYMUNE^®^	CEVA Animal Health, Lenexa, KS, USA	Chicken	Killed inactivated *S*. Enteritidis
AviPro^®^ Megan^®^ Vac 1	ELANCO, Greenfield, IN, USA	Chicken	Live metabolic drift mutant strain of *S*. Enteritidis
Nobilis^®^ Salenvac T	MSD Animal Health, Rahway, NJ, USA	Chicken	Formalin-killed cells of *S*. Enteritidis PT4 & *S*. Typhimurium DT104
Salmoporc^®^	CEVA Animal Health, Lenexa, KS, USA	Swine	Live-attenuated *S*. Typhimurium
Enterisol^®^ *Salmonella*	Boehringer Ingelheim Animal Health, Ingelheim am Rhein, Germany	Swine	Live-attenuated *S*. Typhimurium and *S.* Choleraesuis
BIOSUIS SALM^®^	Animal Health Distributors, Carlow, Ireland	Swine	Formalin-killed *S.* Typhimurium, Derby, Infantis
ARGUS^®^	Merck Animal Health, Rahway, NJ, USA	Swine	Live-attenuated *S*. Choleraesuis
Autogenous Bio One Salmonella^®^	Armor Animal Health, Cortland, NY, USA	Swine	Killed *S*. Cerro, *S*. Heidelberg, *S*. Dublin, and *S*. Typhimurium
Salvexin^®^+B	MSD Animal Health, Rahway, NJ, USA	Sheep/cattle	Killed *S*. Bovismorbificans, *S*. Hindmarsh, *S*. Typhimurium and *S*. Brandenburg
Endovac-Bovi^®^	Animal Health Supply, El Paso, TX, USA	Sheep/cattle	Mutant-attenuated *S*. Typhimurium bacterin
Salmonella Vetovax™ SRP^®^	Veto quinol, Princeville, Canada	Sheep/cattle	killed *S*. Newport

**Table 2:** Inclusion and exclusion criteria in this study.

	Inclusion Criteria	Exclusion Criteria
Process	Primary	Primary
**Screening**	Vaccine studies conducted in different animal models (mice, chicken, swine, bovines, and caprine)	Review articles and guidelines
	Primary research studies containing vaccinated and unvaccinated groups	Non-vaccine studies, non-challenge studies or invitro studies, non-*Salmonella* Studies
	Information on conventional and reverse vaccinology approaches, vaccines and vaccination protocols provided	Non-English
	Evaluation and data of vaccine efficacy provided in	Unable to access the full text of papers
	English language	Vaccines conducted in clinical trials
**Eligibility**	Vaccine studies conducted in different animal models	Studies evaluated immune response alone without an effect of *Salmonella* colonization after challenge
	Studies described the levels of *Salmonella* loads in cecal and fecal contents after vaccination and challenge	Studies evaluated the adjuvant efficacy alone or non-*Salmonella* antigens
	Studies described immune responses after vaccination and challenge	Studies that were unable to estimate *Salmonella* loads

**Table 3:** A detailed overview of *Salmonella* vaccine antigens identified in this review.

	Vaccine Type	Role of Antigen in *Salmonella* Colonization	References
	Bacterin	Killed-whole bacterial cells (multiple antigens) used for immunization	[24,25,26]
**Conventional Approach**	histidine-adenine auxotrophic	Adhesion	[27,28]
	surface-exposed lipoprotein A	Adhesion	[29,30]
	Outer membrane proteins	Adhesion and invasion	[31,32,33,34]
	Flagellar Proteins (*fliC*)	Motility and adherence	[35,36]
	Whole-cell lysate	Adhesion	[15,37,38]
	Capsular polysaccharide (CPS)	Serum resistance	[39,40]
	ABC-Type multidrug efflux pump (MacAB)	Multidrug efflux system	[41]
	flagellar hook-associated proteins (*fliD*)	Adhesion	[42,43,44]
	Hypothetical protein	Protein–Protein interactions	[45,46]
	Peptidoglycan Recognition Protein PGLYRP2	Maintenance of cell wall	[30]
	Crude-cell lysate	Adhesion	[47,48]
**Reverse Vaccinology Approach**	Multi epitope	Adhesion and invasion	[49,50]
	Single peptide	Adhesion	[51,52]

## Data Availability

All data relevant to this work are available from the corresponding author upon request.

## Supplementary Materials

The following supporting information can be downloaded at: https://www.mdpi.com/article/10.3390/vaccines12091067/s1. Table S1: Composition of vaccines, *Salmonella* serovars used as a challenge and effect of vaccines on bacterial load on organs after challenge infection; Table S2: Summary of the vaccine efficacy, safety, and immune responses from the eligible trials at the end of the study.
