# Supplementary material for: The Evolution of Vaccines Development across Salmonella Serovars among Animal Hosts: A Systematic Review
Source: Vaccines (Basel). 2024 Sep 18;12(9):1067. doi: 10.3390/vaccines12091067 (PMC11435802; doi:10.3390/vaccines12091067)
Supplement: Supplementary file 1 [file vaccines-12-01067-s001.zip › vaccines-3155326-supplementary.pdf]

Supplementary Table S1. Composition of vaccines, *Salmonella* serovars used as a challenge and effect of vaccines on bacterial load on organs after challenge infection.

	Vaccine Type	Vaccine Formulations, Antigens	Vaccine dose and route of administration	Age at Vaccination (week)	Country	Strain Used for vaccine antigen preparation	Bacterial Challenge Strain (Dose (log10 CFU)	Age at Challenge (weeks)	Age of Animals at the end of experiment (Weeks)	Total No. of animals used	Reduction in Levels of *Salmonella* Colonization Reported	References
Mice	Live Attenuated	Rough attenuated with MOPS and antibiotics	@10^7 CFU/mouse Orally with booster	3 & 6	USA	*S.* Typhimurium	*S.* Typhimurium @ 10^5 CFU/mice Orally	*9*	15	28	Significant Reduction	-1
		Mutant attenuated, expressing *optA, optB*, Lawsonia flagellin (*LfliC*), and Lawsonia hemolysin (*Lhly*) antigens	@ 2.5 × 10^6 CFU/ animal Orally with booster	4 & 6	South Korea	*S.* Typhimurium	*S.* Typhimurium @ 2x10^9 CFU/mice Orally	*7*	13	60	Significant Reduction	-2
		Mutant attenuated with *hupA* and *hupB* gene deletion	@ 2 × 10^9 CFU/animal/ Orally with booster	9&10	Brazil	*S. enterica*	*S.* Enteritidis @1 × 10^7 CFU Orally	*11*	11	33	Significant Reduction	-3
		Mutant attenuated, with guanine biosynthesis (*guaBA*) and regulatory protease (*clpX*) gene deletion	@10^9 CFU/animal Orally with booster	8 and 11	USA	*S.* Newport	*S.* Newport @4 × 10^7 intraperitoneally	*15*	19	25	Significant Reduction	-4
		Recombinant attenuated, containing aspartate semialdehyde dehydrogenase (Asd)-based balanced-lethal vector-host system and O antigen gene cluster	@10^9 CFU/animal Orally with booster	4 and 8	China	*S.* Typhimurium, *S.* Newport	*S.* Typhimurium, *S.* Newport @ 10^7 each Orally	*9*	13	32	Significant Reduction	-5
		Mutant attenuated with *lpp* gene (*lppA* and *lppB*) deletion	@3 × 10^3 CFU/animal Orally with booster	12 and 13	USA	*S.* Typhimurium	*S.* Typhimurium @ 1 × 10^8 Orally	*15*	19	40	Significant Reduction	-6

		Mutant attenuated with *guaBA* and *clpX* deletion	10^9 CFU/animal Orally	9	USA	*S. Typhimurium, S. Enteritidis*	*S.* Dublin, *S.* Reading, *S.* Java @ 3.6-4.6 × 10^8 Orally	*19*	22	26	Significant Reduction	-7
		Mutant attenuated, with deletion of virulence genes (*phoP* or *aroA*)	10^8 CFU/animal Orally with booster	9,10 and 11	Japan	*S.* Typhimurium	*S.* Typhimurium @5 × 10^8 CFU Orally	*15*	17	45	Non-Significant reduction	-8
		Mutant attenuated with TTSS-2 deficient	@ 10^3 CFU/animal Orally	9	India	*S.* Typhimurium	*S.* Typhimurium @5 × 10^7 CFU Orally	*12*	16	35	Significant Reduction	-9
		Mutant attenuated with *yncD* virulence gene deletion	@ 2 × 10^9 CFU/animal with 7% (w/v) porcine gastric mucin, intraperitoneal (IP)	8	China	*S.* Typhi	*S.* Typhi @ 10^{4-7} CFU/ml Orally	*12*	8	15	Non-Significant reduction	-10
		Mutant attenuated with *hfq* gene deletion	@10^8 CFU/mouse Orally with booster	8,9 and 10	India	*S.* Typhimurium	*S.* Typhimurium @ 10^7 CFU Orally	*11*	16	20	Significant Reduction	-11
		Mutant attenuated, with *RpoS* and *Cys* deletion	@10^9 CFU/mouse Intranasal	3	USA	*S. Typhi, S. ParatyphiA*	*S.* Typhi, *S.* Paratyphi A @10^9 CFU Orally	*5*	9	24	Significant reduction	-12
		Mutant attenuated lacking zinc transporter ZnuABC	@2 × 10^7 CFU/animal Orally with booster	8 and 9	Italy	*S.* Typhimurium	*S.* Typhimurium @ 2 × 10^8 CFU Intragastrically	*10*	14	30	Non-Significant reduction	-13
		Mutant attenuated with *lon* and *cpxR* genes deletion	@2 × 10^{11} CFU/animal Orally with booster	6 and 7	South Korea	*S.* Typhimurium	*S.* Typhimurium @ 2 × 10^8 CFU Orally	*8*	9	105	Significant reduction	-14
		Recombinant attenuated with O-antigen deficient	1×10^{10} CFU/ animals Orally and Intraperitoneal	7	Korea	*S.* Typhimurium	*S.* Typhimurium @ 1× 10^{10} CFU Orally	*11*	13	40	Significant reduction	-15
		Mutant attenuated with *ruvB* gene deletion	@10^7 CFU/animal Orally with booster	6 and 7	Korea	*S.* Typhimurium	*S.* Typhimurium @ 1× 10^7 CFU Orally	*9*	11	24	Significant reduction	-16
		Recombinant *SpaO* and H1 attenuated strain pre-mixed with aluminum hydroxide	@10^8 CFU/animal Orally with booster	9 and 10	China	*S. Typhi, S. ParatyphiA*	*S. Typhi, S. ParatyphiA* @ 1.5 × 10^8 Intraperitoneal	*11*	13	40	Significant reduction	-17
		Mutant attenuated with Δ*lppAB* Δ*msbB* gene deletion	@10^{3-8} CFU/animal Orally with booster	8	USA	*S.* Typhimurium	*S.* Typhimurium @ 1× 10^{4-7} CFU Orally	*10*	11	20	Significant reduction	-18
		Mutant attenuated with *rpoS, phoP* gene deletion	@2x10^9 CFU/animal, Intranasally	4	South Korea	*S.*Typhi	*S.*Typhi @ 1 × 10^3 CFU in 5% hog gastric mucin, Intraperitoneal	*7*	8	16	Significant reduction	-19
		pmrG-HM-D mutant attenuated	1×10^4 and 10^7 CFU/ animals Orally and IP with booster	6 and 7	India	*S.* Typhimurium	*S.* Typhimurium @ 1× 10^3 CFU Orally	*9*	11	18	Significant reduction	-20

		Mutant attenuated with *aroA* gene deletion	@ $1–2x10^5$ CFU/animal, Orally	1,2, and 4	USA	*S.* Typhimurium	*S.* Typhimurium @ $1\times 10^{5-9}$ CFU Orally	*7*	9	20	Significant reduction	-21
		Rough attenuated	@ $1x10^7$ CFU/animal, Orally	10	Italy	*S.* Abortusovis, *S.* Typhimurium	*S.* Abortusovis @ 1×10^9 CFU Orally	*14*	18	20	Significant reduction	-22
		Rough attenuated	@ $5x10^7$ CFU/animal, Orally	1	Switzerland	*S.* Typhimurium	*S.* Typhimurium @ $5x10^{10}$CFU Intraperitoneal	*4*	8	32	No effect	-23
		Rough attenuated	@1×10^9 CFU/animal, Orally	4	USA	*S.* Typhimurium	*S.* Typhimurium @ 2×10^6 to 5×10^6 CFU Orally & Intraperitoneal	*6*	9	28	No effect	-24
		Crude cell lysate, conjugated capsular protein (Vi polysaccharide) with aluminum phosphate adjuvant	@ 1 mg/10 µg/mouse IM with booster	2,4, and 6	Australia	*S.*Typhi	*S.* Typhi @ 1×10^7 CFU Orally	*7*	11	32	Non-significant reduction	-25
		Crude cell lysate with outer membrane vesicles (OMVs)	@5 µg/mice, IP with booster	3 and 6	India	*S.* Typhimurium & *S.* Enteritidis	*S.* Typhimurium *& S.* Enteritidis @ 1×10^7 CFU Orally	*9*	13	24	c	-26
		Crude cell lysate with outer membrane protein C (OmpC) conjugated with Vi polysaccharide	@ 5 µg Vi and 11.8 µg OmpCp, subcutaneous	4	USA	*S.* Typhi	*S.* Typhi @1×10^7 CFU Orally	*6*	8	18	Significant reduction	-27
	Subunit vaccine	Crude cell lysate with core and O-polysaccharide (COPS) conjugated to flagellin protein (FliC)	@ 2.5 µg/ mouse, IM with booster	0,2 and 4	USA	*S.* Typhimurium	*S.* Typhimurium @ $5x10^2$ CFU Intraperitoneal	*6*	14	24	Non-significant reduction	-28
		Recombinant Heat Shock Protein (rHsp60) derived from gram-negative bacterial with incomplete Freund's adjuvant	@10 µg/ mouse, S.C. with booster	6	Poland	*S.* Enteritidis	*S.* Enteritidis @ 2×10^6 CFU Orally	*12*	18	88	Significant reduction	-29
		Crude cell lysate with core and O-polysaccharide (COPS) conjugated to flagellin protein (*FliC*)	@ 2.5 µg/ mouse, IP with booster	4 and 6	USA	*S.* Newport	*S.* Newport @ 6×10^7 CFU Intraperitoneal	*12*	16	40	Significant reduction	-30

		Whole-cell lysate, total protein with lipopolysaccharides (LPS)	@ 20 µg/ mouse, orally with booster	0,2, and 4	China	*S*. Enteritidis	*S*. Enteritidis @ 5 × 10^6 CFU Orally	*8*	11	N/A	Significant reduction	-31
		Crude cell lysate with Outer Membrane Proteins (OMPs) with lipopolysaccharides (LPS)	@10 µg/mice, intranasal with booster	2 and 4	China	*S*. Typhimurium	*S*. Typhimurium @ 10^5 CFU Orally	*10*	13	40	Non-significant reduction	-32
		Crude cell lysate with r*PagN*, an outer membrane protein	@100 µg/mice, IP with booster	6 and 9	China	*S*. Typhimurium	*S*. Typhimurium @1 × 10^6 CFU Orally	*10*	11	35	Significant reduction	-33
		Crude cell lysate with Vi polysaccharide conjugated with outer membrane protein (rP40), in combination with E. coli heat-labile toxin adjuvant (LT)	11 µg/mice, SC and intranasal	0, and 3	UK	*S*. Typhimurium	*S*. Typhimurium @2 × 10^8 CFU Orally	*7*	14	50	Significant reduction	-34
		0-specific capsular polysaccharide conjugated with tetanus toxoid (0-SP-TT)	@ 11 µg/mice, SC	6	USA	*S*. Typhimurium	*S*. Typhimurium @10^6 CFU Orally	*10*	12		No effect	-35
	Inactivated, Killed	Whole-cell lysate encapsulated with nanoparticles	@ 30 µg/ mouse, IP with booster	6 and 8	Spain	*S*. Enteritidis	*S*. Enteritidis @ 1.5 × 10^2 CFU Orally	*12*	17		Significant reduction	-36
		Hot-saline extracts and formalin-inactivated bacterin with (ΔwaaL) or deep-defective (Δgal) LPS-Core, in combination with EDA	@ 40 µg/ mouse, IP with booster	10 and 12	Spain	*S*. Enteritidis	*S*. Enteritidis @ 2.5 × 10^2 CFU Orally	*14*	15	30	Significant reduction	-37
		1% Formalin inactivated with aluminum hydroxide adjuvant	@1 × 10^9 CFU/ animal Orally	2 and 3	South Korea	*S*. Typhimurium	*S*. Typhimurium @ 2 × 10^8 CFU Orally	*7*	9	80	Significant reduction	-14
		Heat-killed phenol-preserved and acetone-killed	@ 15 µg/ mouse, orally with booster	6 and 8	UK	*S*. Typhimurium	*S*. Typhimurium @ 3x10^4 CFU Orally	*10*	12	280	Non-significant reduction	-38
	Bacterial Ghost vaccine	Bacterial ghost cells with the recombinant fusion protein	@5 mg/ mouse, orally with booster	6 and 8	South Korea	*S*. Typhimurium	*S*. Typhimurium @ 2x10^8 CFU Orally	*10*	12	60	Significant reduction	-39

		Bacterial ghost produced the expression of lysis gene E from bacteriophage PhiX174 Neisseria gonorrhoeae porin B (*porB*)	@1 mg/ mouse, orally with booster	6 and 8	China	*S.* Enteritidis	*S.* Enteritidis @ 1 × 10^9 CFU Orally	*10*	15	40	Significant reduction	-40
		Bacterial ghost lysed by the recombinant lysozyme-PMAP36 with fusion protein	@ 2.5 mg/ mouse, IM	10	South Korea	*S.* Typhimurium	*S.* Typhimurium @ 2x10^8 CFU Orally	*16*	18	36	Significant reduction	-41
		Bacterial ghost cassette comprising the PhiX 174 Elysis gene	@ 100 µl/ mouse, IM with booster	4 and 8	South Korea	*S.* Typhi	*S.* Typhi @ 1x10^9 CFU Orally	*10*	12	50	Significant reduction	-42
	Recombinant Vaccine	Recombinant SspH2-EscI fusion protein using the promotor of SspH2, X4550(pYA3334-P-SspH2-EscI) with PBS as vehicle	@ 25 µg/ mouse, orally with booster	6 nd 9	China	*S.* Typhimurium	*S.* Typhimurium @ 1x10^5 CFU Orally	*12*	18	24	Significant reduction	-43
	Subcellular Vaccines	Bacterial surface components enriched hot saline extracts (HE)	@ 30 µg/ mouse, IP	10	Spain	*S.* Enteritidis	*S.* Enteritidis @ 1.6 × 10^2 CFU Orally	*12*	15	30	Significant reduction	-44
	Reverse Vaccinology approach	Recombinant outer membrane protein (FliK, BcsZ, FhuA and FepA) with adjuvant	@ 30 ng/ mouse, IM	4	China	Multi strains	*S.* Typhimurium @ 6.4x10^5 CFU Orally	*6*	8	24	Significant reduction	-45
		Recombinant steD protein identified through reverse vaccinology with complete Freund's adjuvant	@ 30 µg/ mouse, IP with booster	3 and 4	Iran	*S.* Typhi	*S.* Typhi @ 1x10^8 CFU Orally	*6*	7	32	Significant reduction	-46
Chicken	Live Attenuated	Mutant attenuated with deletion of genes related to lipid A	@ 1 × 10^7 cells/chicken, IM with booster	1	Korea	*S.* Gallinarum	*S.* Gallinarum @ 1 × 10^6 IM	*4*	5	200	Significant reduction	-47
		Rough attenuated suspended in gel-diluent	@ 1 × 10^7 CFU/animal Orally	1	Australia	*S.* Typhimurium	*S.* Typhimurium @ 1x10^7 CFU Orally	*5*	10	180	Non-significant reduction	-48
		Mutant attenuated with *rfbG* gene deletion	@ 5 × 10^7 CFU/animal Orally	2 and 4	China	*S.* Enteritidis	*S.* Enteritidis @ 7× 10^8 CFU Orally	*6*	6	75	Significant reduction	-49

			Recombinant temperature-sensitive attenuated	@ 2 × 10^7 CFU/animal Orally and IM	4 and 7	South Korea	*S*. Enteritidis	*S*. Enteritidis @ 2× 10^8 CFU Orally	*9*	9	60	Non-significant reduction	-50
			Rough attenuated	@ 2 × 10^7 CFU/animal S/C	16,18,30 and 42	Bangladesh	*S*. Gallinarum	*S*. Gallinarum @ 4 × 10^7 IM	*21*	54	60	Significant reduction	-51
			Mutant attenuated with three virulence-related genes (*lon, cpxR*, and *rfaL*) deletions	@ 1 × 10^6 CFU/bird orally	1 and 2	South Korea	*S*. Gallinarum	*S*. Gallinarum @ 1 × 10^6 Orally	*8*	10	80	Significant reduction	-52
			Mutant attenuated with *srfA* deletion	@ 4 × 10^7 CFU/bird Orally	2	China	*S*. Pullorum	*S*. Pullorum @ 1 × 10^9 IM	*4*	6	120	Significant reduction	-53
			Mutant attenuated with protein tyrosine phosphatase (*SptP*) gene deletion	@ 1 × 10^{7-9} CFU/animal Orally	1	China	*S*. Enteritidis	*S*. Enteritidis @ 5× 10^8 CFU Orally	*3*	4	80	Non-significant reduction	-54
			Mutant attenuated encapsulated with alginate-coated chitosan microparticles	@ 1 × 10^8 CFU/animal Orally	4	Nigeria	*S*. Gallinarum	*S*. Gallinarum @ 1.2 × 10^8 Orally	*61*	26	81	Non-significant reduction	-55
			Mutant attenuated with deletion of two genes, *aroA* and *serC*	@ 3 × 10^7 CFU/animal Orally	1	Brazil	*S*. Typhimurium	*S*. Heidelberg @ 1x10^9 CFU Orally	*3*	4	81	Significant reduction	-56
			Rough Mutant with *spiC* deletion	@ 3.8 × 10^6 CFU/animal Orally	2	China	*S*. Pullorum	*S*. Pullorum, *S*. Gallinarum *and S*. Enteritidis @ 1x10^9 CFU each Orally	*3*	5	40	Significant reduction	-57
			Mutant attenuated with double deletion of Δ*lon*, Δ*cpxR* genes	@ 1 × 10^9 CFU/animal Orally or IM	1 and 7	South Korea	*S*. Montevideo, S.Enteritidis	*S*. Montevideo @ 1x10^9 CFU Orally	*3*	15	40	Significant reduction	-58
			Mutant attenuated with deletion of *Salmonella* pathogenicity island 1 (SPI1) and replaced with a kanamycin resistance gene cassette (SPI1: Kan mutation)	@ 1 × 10^6 CFU/animal Orally	16	Czech Republic	*S*. Enteritidis, *S*. Typhimurium and *S*. Infantis	*S*. Enteritidis, *S*. Typhimurium and *S*. Infantis, *S*. Agona, *S*. Dublin and *S*. Hadar @ 3 × 10^7 CFU, orally	*3*	22	42	Significant reduction	-59
			Mutant attenuated with *cobS* and *cbiA* gene deletion	@ 1 × 10^8 CFU/animal Orally	3	Brazil	*S*. Gallinarum	*S*. Gallinarum @ 1 × 10^9 Orally	*7*	11	60	Significant reduction	-60

		Rough attenuated	@ 1×10^8 CFU/animal IM	1	Germany	*S.* Enteritidis	*S.* Enteritidis @ 2×10^5 CFU Orally	*2*	3	32	Significant reduction	-61
		Mutant attenuated with deletion of *rpoS*, *hmp*, and *ssrAB* of type III secretion system (T3SS)	@ 1×10^8 CFU/animal Orally	3	South Korea	*S.* Gallinarum	*S.* Gallinarum @ 4×10^8 Orally	*6*	9	48	Significant reduction	-62
		Mutant attenuated with deletion of hilA, *ssrA* and *fliG* genes	@ 1×10^8 CFU/animal Orally	3 and 6	Belgium	*S.* Enteritidis	*S.* Enteritidis @ 1×10^9 CFU Orally	*10*	15	64	Significant reduction	-63
		Mutant attenuated with deletion of *crp*, *rfc* and *rfaH* genes	@ 1×10^8 CFU/animal Orally	3 and 15	USA	*S.* Gallinarum	*S.* Gallinarum, *S.* Enteritidis @ 1×10^{10} Orally	*10*	16	40	Non-significant reduction	-64
		Mutant attenuated with double deletion of *phoP fliC* genes	@ 1×10^8 CFU/animal Orally	3 and 4	Germany	*S.* Enteritidis	*S.* Enteritidis @ 1×10^5 CFU Orally	*1*	7	32	Non-significant reduction	-65
		Rough attenuated	@ 1×10^8 CFU/animal Orally	1 and 7	Korea	*S.* Gallinarum	*S.* Gallinarum @ 4×10^8 Orally	*2*	14	40	Non-significant reduction	-66
		SPI1-lon mutant with or without *fliC* gene	@10^7 CFU/animal orally	2	Czech Republic	*S.* Enteritidis	*S.* Enteritidis @ 3×10^7 Orally	*3*	4	60	Non-significant reduction	-67
		Mutant attenuated with deletion of SPI1, SPI2, *aroA* or *phoP*	@10^8 CFU/animal orally	3	Czech Republic	*S.* Enteritidis	*S.* Enteritidis @ 1×10^9 Orally	*4*	5	48	Non-significant reduction	-68
		mutant attenuated with deletion of *aroA* gene administered	@10^9 CFU/animal orally	2	UK	*S.* Enteritidis	*S.* Enteritidis, *S.* Typhimurium @ 1×10^9 each Orally	*4*	6	32	Significant reduction	-69
	Killed, Inactivated	Crude cell lysate containing total OMPs encapsulated with chitosan nanoparticle	@ 20 µg/bird orally with booster	1	USA	*S.* Enteritidis	*S.* Enteritidis @ 1×10^7 each Orally	*2*	3	300	Significant reduction	-70
		Crude cell lysate containing total OMPs mixed with Freunds Incomplete Adjuvant	@ 100 µg/bird orally with booster	3, and 6	India	*S.* Typhimurium	*S.* Typhimurium @ 4x10^8 CFU IM	*15*	20	180	Non-significant reduction	-71
		Whole cell lysate (bacterin) with Aluminum hydroxide adjuvanted,	@250 µg/ bird orally with booster	2,and 10	Netherlands	*S.* Enteritidis, *S.* Typhimurium, *S.* Infantis	*S.* Enteritidis, *S.* Typhimurium, *S. Virchow*, *S.* Hadar, *S. Heidelberg* @ 1x10^{6-8} CFU	*12, and 14*	21	120	Significant reduction	-72

							Orally					
		Crude cell lysate as oil-based	@0.25ml/bird subcutaneously	5 and 22	Japan	*S.* Enteritidis	*S.* Enteritidis @ 1×10^8 each Orally	*10*	25	220	Significant reduction	-73
		Whole cell lysate (bacterin)	@0.1-0.5ml/bird subcutaneously with booster	2, 4 and 10	UK	*S.* Enteritidis, *S.* Typhimurium,	*S.* Enteritidis, *S.* Typhimurium @ 2-5 $x10^6$ CFU Orally	*3 and 5*	16	48	Significant reduction	-74
		Acetone-killed oil-emulsion bacterin	@150 µg/ bird orally with booster	23 and 45	USA	*S.* Enteritidis	*S.* Enteritidis @ 1×10^9 each Orally	*32*	52	400	Non-significant reduction	-75
		Formalin-killed mineral oil and Arlacel A adjuvanted bacterin	@0.1-0.2ml/bird both subcutaneously and orally	1, 2 and 4	USA	*S.* Typhimurium	*S.* Typhimurium @ $1x10^5$ CFU Orally	*3*	8	120	Non-significant reduction	-76
		Outer membrane vesicles (OMVs) suspended in PBS	@50 µg/chick orally	3,10 and 22	India	*S.* Enteritidis, *S.* Typhimurium, *S.* Gallinarum	*S.* Enteritidis, *S.* Typhimurium, *S.* Gallinarum @ 1 $x10^9$ CFU each Orally	*1, and 10*	52	300	Significant reduction	-77
		Outer membrane vesicles (OMVs) suspended in Dulbecco's phosphate-buffered saline (DPBS)	@20 µg OMVs based on protein content in 10 µl DPBS per animal, intraperitoneal with booster	1, and 2	China	*S.* Typhimurium	*S.* Choleraesuis, *S.* Enteritidis, @ 1 $x10^7$ CFU each Orally	*5*	6	40	Non-significant reduction	-78
	Subunit Vaccine	Mannose-conjugated chitosan-modified outer membrane proteins (OMP) and flagellin (FLA)	@10 µg/bird orally with booster	1	USA	*S.* Enteritidis	*S.* Enteritidis @ 5×10^8 each Orally	*3*	5	65	Significant reduction	-79
		Outer membrane proteins (OMPs) and flagellin (F) protein -coated chitosan nanoparticles (CS NPs) (OMPs-F-CS NPs)	@500 µg/bird orally with booster	5,8 and 11	USA	*S.* Enteritidis	*S.* Enteritidis @ 5×10^6 each Orally	*7*	15	34	Significant reduction	-80
		Purified outer membrane protein A (OMPA)	@80 µg /bird orally with booster	2	Japan	*S.* Enteritidis	*S.* Enteritidis @ 1×10^9 each Orally	*3*	5	40	No effect	-81
		Recombinant *FliC* Protein with PBS	@ 100 µg/bird orally with booster	3, and 8	Japan	*S.* Enteritidis	*S.* Enteritidis @ 1×10^{10} each Orally	*12*	15	160	Non-significant reduction	-82

		purified SPI-1 proteins (*PrgI* and *SipD*) or SPI-2 proteins (*SseB* and *SseD*)	@100 µg/bird subcutaneously with booster	2, and 6	Canada	*S.* Enteritidis	*S.* Enteritidis @ 1 × 10^9 each Orally	*8*	13	64	Significant reduction	-83
		iron-regulated outer membrane proteins (IROMPs) mixed with Freund's incomplete adjuvant @	0.05 mg/ml/bird IM with booster	2, and 3	Japan	*S.* Typhimurium	*S.* Enteritidis, @ 1 x10^8 CFU each Orally	*5*	7	120	Significant reduction	-84
		bacterial ghost cells expressing surface proteins (*fliC-fimA*-CD40L)	@2 X 10^8 CFU/bird IM with booster	1, and 7	South Korea	*S.* Enteritidis, *S.* Gallinarum	*S.* Enteritidis, *S.* Gallinarum @ 2 x10^5 CFU each Orally	*10*	13	90	Significant reduction	-85
		Bacterial ghost cells display on the bacterial surface the H1N1 hemagglutinin globular head portion	@10^9 CFU/bird orally and IM with booster	2, and 4	South Korea	*S.* Enteritidis	*S.* Enteritidis, influenza H1N1@ 2 x10^8 CFU Orally	*7*	9	140	Significant reduction	-86
	Bacterial ghost vaccine	Bacterial ghost cells with *spiC* deletion mutant	@10^{7-9} CFU/bird both orally and IM with booster	1,3 and 5	China	*S.* Pullorum	*S.* Pullorum @ 1 x10^9 CFU IM	*2 and 6*	15	120	Significant reduction	-87
		Bacterial ghost carrying heat-labile enterotoxin B subunit (LTB) suspension with Montanide	@10^9 CFU/bird both orally with booster	5	South Korea	*S.* Typhimurium	*S.* Typhimurium @ 1x10^9 CFU Orally	*3*	10	56	Significant reduction	-88
		Bacterial ghost cells with controlled expression of the bacteriophage PhiX174	@10^8 CFU/bird orally, IM, subcutaneously with booster	1	South Korea	with booster	with booster	3	4	120	Significant reduction	-89
	Reverse Vaccinology	Recombinant proteins identified through reverse vaccinology approach	@20 µg diluted in 100 µL PBS mixed with 100 µL Rhizoma Atractylodis Macrocephalae polysaccharides adjuvant	1	China	*S.* Pullorum	*S.* Pullorum @ 1 x10^7 CFU Orally	*2*	3	36	Significant reduction	-90
Swine	Live Attenuated	Mutant attenuated with histidine-adenine auxotrophic genes deletion	@10^8 CFU/animal subcutaneously (SC) with booster	12 and 16	Belgium	*S.* Typhimurium	*S.* Typhimurium @ 10^9 CFU orally	*21*	37	3 farms	Non-Significant reduction	-91

		Mutant attenuated, with deletion of deletions in all genes encoding GGDEF domain proteins and in *rpoS*	@2.8 × 10^9 CFU Orally with booster	1 and 4	Spain	*S*. Enteritidis	*S*. Typhimurium @ 3.4 × 10^9 CFU Orally	*6*	22	60	Significant reduction	-92
		Rough attenuated, with chemically induced mutation adenine/histidine auxotrophic	@5 × 10^8 CFU/animal Orally with booster	1, and 3	Germany	*S*. Typhimurium	*S*. *enterica* 4,[5],12: i: (DT 193) @ 5 × 10^9CFU Orally	*6*	7	40	Significant reduction	-93
		A combination of the roughly attenuated strain lacking Zinc transporter	@ 5 × 10^7CFU/ animal Orally	5	Italy	*S*. Typhimurium<br>*S*. Choleraesuis	*S*. Choleraesuis<br>@5 × 10^8 CFU by oral gavage	*7*	9	18	Non-Significant reduction	-94
		Rough attenuated by lytic bacteriophage activity	@ 3x10^9 CFU/animal Orally	4	UK	*S*. Infantis	*S*. Typhimurium @5 × 10^9 CFU Orally	*5*	6	24	Significant reduction	-95
		LPS-mutant strain with the deletion of the '*rfaJ*' gene	@10^7 – 10^8 CFU/animal Orally	4 and 7	Belgium	*S*. Typhimurium	*S*. Typhimurium @ 10^7 CFU Orally	*10*	15	56	Non-Significant reduction	-96
		Mutant attenuated with *lon*, *asd* and *cpxR* genes deletion	@ 2 × 10^9 CFU/ animal Orally	3 and 7	South Korea	*S*. Typhimurium	*S*. Typhimurium @5 × 10^9 CFU Orally	*8*	13	116	Non-Significant reduction	-97
		Mutant attenuated with deletion of *rpoS* and *phoP* gene	@ 1× 10^9 CFU/ animal Orally with booster	2 and 5	Spain	*S*. Choleraesuis	*S*. Choleraesuis<br>@ 10^8 CFU Orally	*11*	14	32	Non-Significant reduction	-98
		Mutant attenuated with *crp* gene deletion	@ 10^8 CFU/animal orally, intramuscularly (IM) with booster	3 and 4	China	*S*. Choleraesuis	*S*. Choleraesuis @ 4.6 × 109 Orally	*6*	8	40	Significant Reduction	-99
		Mutant attenuated with *cya*, *crp* and *aroA* gene deletion	@3×10^9 CFU/animal orally with booster	2 and 3	UK	*S*. Typhimurium, *S*. Enteritidis	*S*. Typhimurium<br>@ 10^6 CFU orally	*4*	6	18	Significant Reduction	-100
	Inactivated, Killed	Inactivated by Formalin and suspended into fenicated physiological serum	@ 10^8 CFU/animal Orally with booster	3, and 5	Brazil	*S*. Typhimurium, *S*. Choleraesuis<br>, *P. Multocida*	*S*. Typhimurium<br>@ 10^8 CFU orally	*7*	9	40	Significant Reduction	-101
		Inactivated by 0.8% formaldehyde without adjuvant	@ 2 × 10^9 CFU/ml/animal Intramuscularly (IM)	3	Italy	*S*. Typhimurium<br>*S*. Choleraesuis	*S*. Choleraesuis<br>@5 × 10^8 CFU by oral gavage	*5*	7	24	Non-Significant reduction	-94

		Crude cell lysate, inactivated with 37% formaldehyde (bacterin) with Al $(OH)_3$ adjuvant	@5 × 10^9 CFU/animal Orally with booster	4, and 6	Spain	*S*. Typhimurium	*Salmonella* spp. @ 10^7 CFU Orally	*11*	13	34	Significant Reduction	-102
		Inactivated with 1% formaldehyde with Al $(OH)_3$ adjuvant	@ 2 × 10^{10} CFU/animal Orally	6	South Korea	*S*. Typhimurium	*S*. Typhimurium @5 × 10^9 CFU Orally	*11*	13	115	Non-Significant reduction	-103
Bovine	Killed, Inactivated	Whole bacterial cell lysate suspended in PBS	@2 ml/animal subcutaneously	1, 3 and 5	USA	*S*. Newport	*S*. Newport, *S*. Montevideo, *S*. Anatum @ 4.6 x10^7 CFU each SC	*12*	18	40	Significant reduction	-104
		Formalin-killed strain suspended in PBS	@2 ml/animal Intramuscularly	3 and 6	Sweden	*S*. Typhimurium	S. Typhimurium @ 1 x10^8 CFU, IM	16	25	20	Non-significant reduction	-105
Caprine	Live attenuated	Rough attenuated	@10^9 CFU/ animal orally	4 and 10	Spain	*S.* Abortusovis	*S*. Abortusovis @ 5 × 10^6 CFU, SC	*15*	18	60	Significant reduction	-106
		Mutant attenuated with *aroA gene* deletion	@ 1.6 × 10^9 CFU/animal Orally with booster SC	6 and 12	Italy	*S.* Abortusovis<br>*S*. Typhimurium	*S*. Abortusovis @ 1× 10^9 CFU, SC	*18*	28	50	Significant reduction	-23

Supplementary Table S2. Summary of the vaccine efficacy, safety, and immune responses from the eligible trials at the end of study.

	Vaccine Type	Vaccine Formulations, Antigens	Vaccine dose and route of administration	Bacterial Challenge Strain (Dose (log10 CFU)	Safety Profile	Immunology	Efficacy (%)	References
Mice	Live Attenuated	Rough attenuated with MOPS and antibiotics	@10^7 CFU/mouse Orally with booster	*S.* Typhimurium @ 10^5 CFU/mice Orally	*No AEs*	Non-significant increase in IgG response	75	[1]
		Mutant attenuated, expressing *optA*, *optB*, Lawsonia flagellin (*LfliC*), and Lawsonia hemolysin (*Lhly*) antigens	@ 2.5 × 10^6 CFU/ animal Orally with booster	*S.* Typhimurium @ 2x10^9 CFU/mice Orally	*No AEs*	Significant increase in IgG, mucosal IgA responses and interferon-γ responses	100	[2]
		Mutant attenuated with *hupA* and *hupB* gene deletion	@ 2 × 10^9 CFU/animal/ Orally with booster	*S.* Enteritidis @1 × 10^7 CFU Orally	*Mild AEs*	Significant increase in IgG and IgA antibodies against Salmonella and TH1-related cytokines (IFN-γ and TNF-α)	80	[3]
		Mutant attenuated, with guanine biosynthesis (*guaBA*) and regulatory protease (*clpX*) gene deletion	@10^9 CFU/animal Orally with booster	*S.* Newport @4 × 10^7 intraperitoneally	*No AEs*	Significant increase in IL-4 and IFN-γ	100	[4]
		Recombinant attenuated, containing aspartate semialdehyde dehydrogenase (Asd)-based balanced-lethal vector-host system and O antigen gene cluster	@10^9 CFU/animal Orally with booster	*S.* Typhimurium,<br><br>*S.* Newport @ 10^7 each Orally	*No AEs*	Significant increase in *Salmonella*-specific IgG response	100	[5]
		Mutant attenuated with *lpp* gene (*lppA* and *lppB*) deletion	@3 × 10^3 CFU/animal Orally with booster	*S.* Typhimurium<br><br>@ 1 × 10^8 Orally	*No AEs*	Significant increase in IgG, IL-6 and IL-17A	100	[6]\
		Mutant attenuated with *guaBA* and *clpX* deletion	@10^9 CFU/animal Orally with booster	*S.* Dublin, *S.* Reading, *S.* Java @ 3.6-4.6 × 10^8 Orally	*No AEs*	Non-significant increase in *Salmonella*-specific IgG	81-91 against different sovars	[7]

						response		
		Mutant attenuated, with deletion of virulence genes (*phoP* or *aroA*)	@ 10^8 CFU/animal Orally	*S.* Typhimurium @5 × 10^8 CFU Orally	*No AEs*	Non-significant increase in *Salmonella*-specific IgG and IgA response	50	[8]
		Mutant attenuated with TTSS-2 deficient	@ 10^3 CFU/animal Orally	*S.* Typhimurium @5 × 10^7 CFU Orally	*No AEs*	Significant increase in IgG, mucosal IgA responses	100	[9]
		Mutant attenuated with *yncD* virulence gene deletion	@ 2 × 10^9 CFU/animal with 7% (w/v) porcine gastric mucin, intraperitoneal (IP)	*S.* Typhi @ 10^{4-7} CFU/ml Orally	*No AEs*	Significant increase in antibody responses	33.3-100	[10]
		Mutant attenuated with *hfq* gene deletion	@10^8 CFU/mouse Orally with booster	*S.* Typhimurium @ 10^7 CFU Orally	*No AEs*	Significant increase in serum IgG and secretory-IgA and CD4+ T lymphocytes	100	[11]
		Mutant attenuated, with *RpoS* and *Cys* deletion	@10^9 CFU/mouse Intranasal	*S.* Typhi, *S.* Paratyphi A @10^9 CFU Orally	Mild *AEs*	No effect	80	[12]
		Mutant attenuated lacking zinc transporter ZnuABC	@2 × 10^7 CFU/animal Orally with booster	*S.* Typhimurium @ 2 × 10^8 CFU Intragastrically	*No AEs*	Non-significant increase in IFN-γ	100	[13]
		Mutant attenuated with *lon* and *cpxR* genes deletion	@2 × 10^{11} CFU/animal Orally with booster	*S.* Typhimurium @ 2 × 10^8 CFU Orally	*No AEs*	Significant increase in serum IgG and secretory IgA titers and levels of serum IFN-γ, IL-4, TNF-α, IL-12	100	[14]
		Recombinant attenuated with O-antigen deficient	1×10^{10} CFU/ animals Orally and Intraperitoneal	*S.* Typhimurium @ 1× 10^{10} CFU Orally	*No AEs*	Significant increase in serum IgG and secretory IgA titers and cytokines production	100	[15]
		Mutant attenuated with *ruvB* gene deletion	@10^7 CFU/animal Orally with booster	*S.* Typhimurium @ 1× 10^7 CFU Orally	*No AEs*	Significant increase in total IgG and IFN-γ	100	[16]

		Recombinant *SpaO* and H1 attenuated strain pre-mixed with aluminum hydroxide	@10^8 CFU/animal Orally with booster	*S. Typhi, S. ParatyphiA* @ 1.5 × 10^8 Intraperitoneal	*No AEs*	Significant increase in serum IgG	66.6	[17]
		Mutant attenuated with Δ*lppAB* Δ*msbB* gene deletion	@10^{3-8} CFU/animal Orally with booster	*S.* Typhimurium @ 1× 10^{4-7} CFU Orally	*No AEs*	Significant increase in serum IgG	100	[18]
		Mutant attenuated with *rpoS, phoP* gene deletion	@2x10^9 CFU/animal, Intranasally	*S.*Typhi @ 1 × 10^3 CFU in 5% hog gastric mucin, Intraperitoneal	*No AEs*	Non-significant increase in *Salmonella*-specific IgG response	87	[19]
		pmrG-HM-D mutant attenuated	1×10^4 and 10^7 CFU/ animals Orally and IP with booster	*S.* Typhimurium @ 1× 10^3 CFU Orally	*No AEs*	Significant increase in CD8+ and CD4+ T cell responses	100	[20, 21]
		Mutant attenuated with *aroA* gene deletion	@ 1–2x10^5 CFU/animal, Orally	*S.* Typhimurium @ 1× 10^{5-9} CFU Orally	*No AEs*	Significant increase in *Salmonella*-specific IgG response	100	[21]
		Rough attenuated	@ 1x10^7 CFU/animal, Orally	*S.* Abortusovis @ 1× 10^9 CFU Orally	*No AEs*	Non-significant increase in IL-2, IL-4, IFN-γ	70	[22, 23]
		Rough attenuated	@ 5x10^7 CFU/animal, Orally	*S.* Typhimurium @ 5x10^{10}CFU Intraperitoneal	Mild to moderate *AEs*	No effect	No effect	[23]
		Rough attenuated	@1× 10^9 CFU/animal, Orally	*S.* Typhimurium @ 2 × 10^6 to 5 × 10^6 CFU Orally & Intraperitoneal	Mild to moderate *AEs*	Non-significant increase in *Salmonella*-specific IgG response	87	[24]
	Subunit vaccine	Crude cell lysate, conjugated capsular protein (Vi polysaccharide) with aluminum phosphate adjuvant	@ 1 mg/10 µg/mouse IM with booster	*S.* Typhi @ 1× 10^7 CFU Orally	*No AEs*	Significant increase in *Salmonella*-specific IgG response	70	[25]
		Crude cell lysate with outer membrane vesicles	@5 µg/mice, IP with booster	*S.* Typhimurium *& S.* Enteritidis @ 1× 10^7 CFU	*No AEs*	Significant increase in serum	100	[26]

		(OMVs)		Orally		IgG		
		Crude cell lysate with outer membrane protein C (OmpC) conjugated with Vi polysaccharide	@ 5 µg Vi and 11.8 µg OmpCp, subcutaneous	*S.* Typhi @1× 10^7 CFU Orally	*No AEs*	Significant increase in serum IgG and T helper cells	100	[27]
		Crude cell lysate with core and O-polysaccharide (COPS) conjugated to flagellin protein (*FliC*)	@ 2.5 µg/ mouse, IM with booster	*S.* Typhimurium @ $5x10^2$ CFU Intraperitoneal	*No AEs*	Significant increase in antibody response	100	[28]
		Recombinant Heat Shock Protein (rHsp60) derived from gram-negative bacterial with incomplete Freund's adjuvant	@10 µg/ mouse, S.C. with booster	*S.* Enteritidis @ 2×10^6 CFU Orally	*No AEs*	Significant increase in serum IgG and IL-6	100	[29]
		Crude cell lysate with core and O-polysaccharide (COPS) conjugated to flagellin protein (*FliC*)	@ 2.5 µg/ mouse, IP with booster	*S.* Newport @ 6×10^7 CFU Intraperitoneal	*No AEs*	Significant increase in serum IgG	60	[30]
		Whole-cell lysate, total protein with lipopolysaccharides (LPS)	@ 20 µg/ mouse, orally with booster	*S.* Enteritidis @ 5×10^6 CFU Orally	*No AEs*	Significant increase in serum IgG	100	[31]
		Crude cell lysate with Outer Membrane Proteins (OMPs) with lipopolysaccharides (LPS)	@10 µg/mice, intranasal with booster	*S.* Typhimurium @ 10^5 CFU Orally	Mild *AEs*	Non Significant increase in Immune responses	80	[32]
		Crude cell lysate with r*PagN*, an outer membrane protein	@100 µg/mice, IP with booster	*S.* Typhimurium @1×10^6 CFU Orally	*No AEs*	Significant increase in Immune responses	80	[33]
		Crude cell lysate with Vi polysaccharide conjugated with outer membrane	@ 11 µg/mice, SC and intranasal	*S.* Typhimurium @2×10^8 CFU Orally	*No AEs*	Significant increase in serum IgG	70	[34]

		protein (rP40), in combination with E. coli heat-labile toxin adjuvant (LT)						
		0-specific capsular polysaccharide conjugated with tetanus toxoid (0-SP-TT)	@ 11 µg/mice, SC	*S*. Typhimurium @10^6 CFU Orally	*No AEs*	Non-Significant increase in serum IgG, IgM	50	[35]
		Whole-cell lysate encapsulated with nanoparticles	@ 30 µg/ mouse, IP with booster	*S*. Enteritidis @ 1.5×10^2 CFU Orally	*No AEs*	Non-Significant increase in serum IgG, IgM	80	[36]
	Inactivated, Killed	Hot-saline extracts and formalin-inactivated bacterin with (ΔwaaL) or deep-defective (Δgal) LPS-Core, in combination with EDA	@ 40 µg/ mouse, IP with booster	*S*. Enteritidis @ 2.5×10^2 CFU Orally	*No AEs*	Significant increase in serum IgG	100	[37]
		1% Formalin inactivated with aluminum hydroxide adjuvant	@1×10^9 CFU/ animal Orally	*S*. Typhimurium @ 2×10^8 CFU Orally	*No AEs*	Significant increase in serum IgG	100	[14]
		Heat-killed phenol-preserved and acetone-killed	@ 15 µg/ mouse, orally with booster	*S*. Typhimurium @ 3×10^4 CFU Orally	*No AEs*	Non-Significant increase in serum IgG and IgM	40	[38]
	Bacterial Ghost vaccine	Bacterial ghost cells with the recombinant fusion protein	@5 mg/ mouse, orally with booster	*S*. Typhimurium @ 2×10^8 CFU Orally	*No AEs*	Significant increase in serum IgG	80	[39]
		Bacterial ghost produced the expression of lysis gene E from bacteriophage PhiX174 Neisseria gonorrhoeae porin B (*porB*)	@1 mg/ mouse, orally with booster	*S*. Enteritidis @ 1×10^9 CFU Orally	*No AEs*	Significant increase in IgG, IgA, IL-1β, IL-6, IL-10 and IL-12p70	80	[40]
		Bacterial ghost lysed by the recombinant lysozyme-PMAP36 with fusion protein	@ 2.5 mg/ mouse, IM	*S*. Typhimurium @ 2×10^8 CFU Orally	*No AEs*	Significant increase in IgG, IL-10 and IFN-γ	75	[41]
		Bacterial ghost cassette comprising	@ 100 µl/ mouse, IM with booster	*S*. Typhi @ 1×10^9 CFU	*No AEs*	Significant increase in	87.5%	[42]

		the PhiX 174 Elysis gene		Orally		CD3+CD4+ T cells, IL-2, IL-6, IL-12, IL-17 and IFN-γ		
	Recombinant Vaccine	Recombinant SspH2-EscI fusion protein using the promotor of SspH2, X4550(pYA3334-P-SspH2-EscI) with PBS as vehicle	@ 25 µg/ mouse, orally with booster	*S*. Typhimurium @ 1x10^5 CFU Orally	*No AEs*	Significant increase in CD4+, CD8+ T cells responses	75	[43]
	Subcellular Vaccines	Bacterial surface components enriched hot saline extracts (HE)	@ 30 µg/ mouse, IP	*S*. Enteritidis @ 1.6 × 10^2 CFU Orally	*No AEs*	Significant increase in IgG, IgA, IL-1β, IL-6, IL-10 and IFN-γ responses	50–60%	[44]
	Reverse Vaccinology approach	Recombinant outer membrane protein (*FliK*, *BcsZ*, *FhuA* and *FepA*) with adjuvant	@ 30 ng/ mouse, IM	*S*. Typhimurium @ 6.4x10^5 CFU Orally	*No AEs*	Non-Significant increase in serum IgG response	30-70	[45]
		Recombinant *steD* protein identified through reverse vaccinology with complete Freund's adjuvant	@ 30 µg/ mouse, IP with booster	*S*. Typhi @ 1x10^8 CFU Orally	*No AEs*	Non-Significant increase in serum IgG and IgM response	70%	[46]
Chicken	Live Attenuated	Mutant attenuated with deletion of genes related to lipid A	@ 1 × 10^7 cells/chicken, IM with booster	*S*. Gallinarum @ 1 × 10^6 IM	*No AEs*	Non-Significant increase in IgA, IgY, CD4+, CD8+,IFN-γ, TNF-α, and IL-1β	50	[47]
		Rough attenuated suspended in gel-diluent	@ 1 × 10^7 CFU/animal Orally	*S*. Typhimurium @ 1x10^7 CFU Orally	Mild AEs	No effect	40	[48]
		Mutant attenuated with *rfbG* gene deletion	@ 5 × 10^7 CFU/animal Orally	*S*. Enteritidis @ 7× 10^8 CFU Orally	Mild AEs	Significant increase in IgG, mucosal IgA responses	100	[49]
		Recombinant temperature-sensitive attenuated	@ 2 × 10^7 CFU/animal Orally and IM	*S*. Enteritidis @ 2× 10^8 CFU Orally	No AEs	Significant increase in antibody and IFN-γ response	100	[50]

		Rough attenuated	@ 2 × 10^7 CFU/animal S/C	*S*. Gallinarum @ 4 × 10^7 IM	No AEs	Significant increase in antibody response	80	[51]
		Mutant attenuated with three virulence-related genes (*lon, cpxR*, and *rfaL*) deletions	@ 1 × 10^6 CFU/bird orally	*S*. Gallinarum @ 1 × 10^6 Orally	No AEs	Significant increase in IFN-γ, IL-2, IL-12, and IL-4	70	[52]
		Mutant attenuated with *srfA* deletion	@ 4 × 10^7 CFU/bird Orally	*S*. Pullorum @ 1 × 10^9 IM	No AEs	Significant increase in IgA, CD4+,CD8+,IFN-γ, TNF-α, and IL-1β	90	[53]
		Mutant attenuated with protein tyrosine phosphatase (*SptP*) gene deletion	@ 1 × 10^{7-9} CFU/animal Orally	*S*. Enteritidis @ 5× 10^8 CFU Orally	No AEs	Significant increase in antibody responses	80	[54]
		Mutant attenuated encapsulated with alginate-coated chitosan microparticles	@ 1 × 10^8 CFU/animal Orally	*S*. Gallinarum @ 1.2 × 10^8 Orally	Mild AEs	Significant increase in IgG and IFN-γ response	100	[55]
		Mutant attenuated with deletion of two genes, *aroA* and *serC*	@ 3 × 10^7 CFU/animal Orally	*S*. Heidelberg @ 1x10^9 CFU Orally	No AEs	NR	100	[56]
		Rough Mutant with *spiC* deletion	@ 3.8 × 10^6 CFU/animal Orally	*S*. Pullorum, *S*. Gallinarum *and S*. Enteritidis @ 1x10^9 CFU each Orally	No AEs	Significant increase in humoral and cellular immune responses	100	[57]
		Mutant attenuated with double deletion of Δ*lon*, Δ*cpxR* genes	@ 1 × 10^9 CFU/animal Orally or IM	*S*. Montevideo @ 1x10^9 CFU Orally	No AEs	Significant increase in plasma IgG, CD4+, IL-6, IL-12,and IFN-γ,	100	[58]
		Mutant attenuated with deletion of *Salmonella* pathogenicity island 1 (SPI1) and replaced with a kanamycin resistance gene	@ 1 × 10^6 CFU/animal Orally	*S*. Enteritidis, *S*. Typhimurium and *S*. Infantis, *S*. Agona, *S*. Dublin and *S*. Hadar @ 3 × 10^7 CFU, orally	No AEs	No effect	70	[59]

		cassette (SPI1: Kan mutation)						
		Mutant attenuated with *cobS* and *cbiA* gene deletion	@ 1 × 10^8 CFU/animal Orally	*S*. Gallinarum @ 1 × 10^9 Orally	Mild AEs	Significant increase in IL-12, and IFN-γ,	100	[60]
		Rough attenuated	@ 1 × 10^8 CFU/animal IM	*S*. Enteritidis @ 2× 10^5 CFU Orally	No AEs	Significant increase in IgG, IL-12, and IFN-γ,	100	[61]
		Mutant attenuated with deletion of *rpoS*, *hmp*, and *ssrAB* of type III secretion system (T3SS)	@ 1 × 10^8 CFU/animal Orally	*S*. Gallinarum @ 4 × 10^8 Orally	Mild AEs	Significant increase in IgG, mucosal IgA responses	80	[62]
		Mutant attenuated with deletion of *hilA*, *ssrA* and *fliG* genes	@ 1 × 10^8 CFU/animal Orally	*S*. Enteritidis @ 1× 10^9 CFU Orally	No AEs	Significant increase in IgG and IFN-γ response	100	[63]
		Mutant attenuated with deletion of *crp*, *rfc* and *rfaH* genes	@ 1 × 10^8 CFU/animal Orally	*S*. Gallinarum, *S*. Enteritidis @ 1 × 10^{10} Orally	No AEs	Significant increase in immunogenic proteins	100	[64]
		Mutant attenuated with double deletion of *phoP fliC* genes	@ 1 × 10^8 CFU/animal Orally	*S*. Enteritidis @ 1× 10^5 CFU Orally	No AEs	Significant increase in antibody response	100	[65]
		Rough attenuated	@ 1 × 10^8 CFU/animal Orally	*S*. Gallinarum @ 4 × 10^8 Orally	No AEs	Significant increase in humoral and cellular immune responses	100	[66]
		SPI1-lon mutant with or without *fliC* gene	@ 1 × 10^6 CFU/animal Orally	*S*. Enteritidis @ 3 × 10^7 Orally	No AEs	Significant increase in antibody responses	90	[67]
		Mutant attenuated with deletion of SPI1, SPI2, *aroA* or *phoP*	@10^8 CFU/animal orally	*S*. Enteritidis @ 1 × 10^9 Orally	Mild AEs	Non-significant increase in IgG, IL-12, IL-17 and IFN-γ	67	[68]
		mutant attenuated with deletion of *aroA*	@10^9 CFU/animal	*S*. Enteritidis, *S*. Typhimurium @	Mild AEs	Significant increase in	70	[69]

		gene administered	orally	1 × 10^9 each Orally		antibody responses		
	Killed, Inactivated	Crude cell lysate containing total OMPs encapsulated with chitosan nanoparticle	@ 20 µg/bird orally with booster	*S*. Enteritidis @ 1 × 10^7 each Orally	No AEs	Significant increase in antigen-specific IgY/IgA and lymphocyte-proliferation	87	[70]
		Crude cell lysate containing total OMPs mixed with Freunds Incomplete Adjuvant	@ 100 µg/bird orally with booster	*S*. Typhimurium @ 4x10^8 CFU IM	No AEs	Significant increase in antibody responses	100	[71]
		Whole cell lysate (bacterin) with Aluminum hydroxide adjuvanted,	@250 µg/ bird orally with booster	*S*. Enteritidis, *S*. Typhimurium, *S. Virchow, S*. Hadar, *S*. *Heidelberg* @ 1x10^{6-8} CFU Orally	No AEs	NR	70-93	[72]
		Crude cell lysate as oil oil-based	@0.25ml/bird subcutaneously	*S*. Enteritidis @ 1 × 10^8 each Orally	Mild AEs	Significant increase in Anti-SE IgG and anti-SE IgA	100	[73]
		Whole cell lysate (bacterin)	@0.1-0.5ml/bird subcutaneously with booster	*S*. Enteritidis, *S*. Typhimurium @ 2-5 x10^6 CFU Orally	No AEs	No effect	68	[74]
		Acetone-killed oil-emulsion bacterin	@150 µg/ bird orally with booster	*S*. Enteritidis @ 1 × 10^9 each Orally	No AEs	No effect	No effect	[75]
		Formalin-killed mineral oil and Arlacel A adjuvanted bacterin	@0.1-0.2ml/bird both subcutaneously and orally	*S*. Typhimurium @ 1x10^5 CFU Orally	No AEs	NR	No effect	[76]
	Subunit Vaccine	Outer membrane vesicles (OMVs) suspended in PBS	@50 µg/chick orally	*S*. Enteritidis, *S*. Typhimurium, *S*. Gallinarum @ 1 x10^9 CFU each Orally	No AEs	Non-significant increase in Igy and mucosal IgA responses	50	[77]
		Outer membrane vesicles (OMVs)	@20 µg OMVs based on protein	*S*. Choleraesuis,	Mild AEs	Significant increase in	70	[78]

		suspended in Dulbecco's phosphate-buffered saline (DPBS)	content in 10 µl DPBS per animal, intraperitoneal with booster	*S.* Enteritidis, @ 1 x10^7 CFU each Orally		antibody response		
		Mannose-conjugated chitosan modified outer membrane proteins (OMP) and flagellin (FLA)	@10 µg/bird orally with booster	*S.* Enteritidis @ 5 × 10^8 each Orally	No AEs	Significant increase in IgG and IFN-γ response	100	[79]
		Outer membrane proteins (OMPs) and flagellin (F) protein coated chitosan nanoparticles (CS NPs) (OMPs-F-CS NPs)	@500 µg/bird orally with booster	*S.* Enteritidis @ 5 × 10^6 each Orally	No AEs	Significant increase in mucosal IgA and IgY response	100	[80]
		Purified outer membrane protein A (OMPA)	@80 µg /bird orally with booster	*S.* Enteritidis @ 1 × 10^9 each Orally	No AEs	Significant increase in anti-OmpA IgG response	100	[81]
		Recombinant *FliC* Protein with PBS	@ 100 µg/bird orally with booster	*S.* Enteritidis @ 1 × 10^{10} each Orally	No AEs	Significant increase in anti-rFliC IgG response	80	[82]
		purified SPI-1 proteins (PrgI and SipD) or SPI-2 proteins (SseB and SseD)	@100 µg/bird subcutaneously with booster	*S.* Enteritidis @ 1 × 10^9 each Orally	No AEs	Non-significant increase in IgG responses	70	[83]
		iron-regulated outer membrane proteins (IROMPs) mixed with Freund's incomplete adjuvant @	0.05 mg/ml/bird IM with booster	*S.* Enteritidis, @ 1 x10^8 CFU each Orally	No AEs	Significant increase in IgG responses	90	[84]
	Bacterial ghost vaccine	bacterial ghost cells expressing surface proteins (*fliC-fimA*-CD40L)	@2 X 10^8 CFU/bird IM with booster	*S.* Enteritidis, *S.* Gallinarum @ 2 x10^5 CFU each Orally	No AEs	Significant increase in IgY, CMI, and cytokine responses	100	[85]
		Bacterial ghost cells display on the bacterial surface the H1N1 hemagglutinin globular head	@10^9 CFU/bird orally and IM with booster	*S.* Enteritidis, influenza H1N1@ 2 x10^8 CFU Orally	No AEs	Significant increase in CD3+CD4+ and CD3+CD8+ T-cell response	100	[86]

		portion						
		Bacterial ghost cells with *spiC* deletion mutant	@10^{7-9} CFU/bird both orally and IM with booster	*S.* Pullorum @ 1 x10^{9} CFU IM	No AEs	Significant increase in IgG responses	100	[87]
		Bacterial ghost carrying heat-labile enterotoxin B subunit (LTB) suspension with Montanide	@10^{9} CFU/bird both orally with booster	*S.* Typhimurium @ 1x10^{9} CFU Orally	No AEs	Significant increase in IgG and IgA responses	100	[88]
		Bacterial ghost cells with controlled expression of the bacteriophage PhiX174	@10^{8} CFU/bird orally, IM, subcutaneously with booster	*S.* Gallinarum @ 1 x106 CFU Orally	No AEs	Significant increase in IgG, IgA and humoral immune responses	100	[89]
	Reverse Vaccinology	Recombinant proteins identified through reverse vaccinology approach	@20 µg diluted in 100 µL PBS mixed with 100 µL Rhizoma Atractylodis Macrocephalae polysaccharides adjuvant Inovo	*S.* Pullorum @ 1 x10^{7} CFU Orally	No AEs	Significant increase in antibody responses	75	[90]
Swine	Live Attenuated	Mutant attenuated with histidine-adenine auxotrophic genes deletion	@10^{8} CFU/animal subcutaneously with booster	*S.* Typhimurium @ 10^{9} CFU orally	No AEs	Significant increase in antibody response	70	[91]
		Mutant attenuated, with deletion of deletions in all genes encoding GGDEF domain proteins and in *rpoS*	@2.8 × 10^{9} CFU Orally with booster	*S.* Typhimurium @ 3.4 × 10^{9} CFU Orally	NR	Significant increase in antibody response	83% during post weaning stage and 41.1% at Slaughtering age	[92]
		Rough attenuated, with chemically induced mutation adenine/histidine auxotrophic	@5 × 10^{8} CFU/animal Orally with booster	*S. enterica* 4,[5],12: i: (DT 193)<br><br>@ 5 × 10^{9}CFU Orally	No AVEs	Significant increase in Salmonella-specific IgA and IgG antibody levels and low IgM levels	82%	[93]
		A combination of the roughly attenuated strain lacking Zinc transporter	@ 5 × 10^{7}CFU/ animal Orally	*S.* Choleraesuis<br><br>@5 × 10^{8} CFU	No AEs	Significant increase in antibody response	75	[94]

				by oral gavage				
		Rough attenuated by lytic bacteriophage activity	@ 3x10^9 CFU/animal Orally	*S*. Typhimurium @5 × 10^9 CFU Orally	No AEs	No effect	No effect	[95]
		LPS-mutant strain with the deletion of the *'rfaJ'* gene	@10^7 – 10^8 CFU/animal Orally	*S*. Typhimurium @ 10^7 CFU Orally	No AEs	Non- Significant increase in antibody response	67%	[96]
		Mutant attenuated with *lon, asd* and *cpxR* genes deletion	@ 2 × 10^9 CFU/ animal Orally	*S*. Typhimurium @5 × 10^9 CFU Orally	No AEs	Significant increase in Salmonella-specific IgA and IgG antibody levels	100	[97]
		Mutant attenuated with deletion of *rpoS* and *phoP* gene	@ 1× 10^9 CFU/ animal Orally with booster	*S*. Choleraesuis<br><br>@ 10^8 CFU Orally	No AEs	Non- Significant increase in antibody response	70	[98]
		Mutant attenuated with *crp* gene deletion	@ 10^8 CFU/animal orally, intramuscularly (IM) with booster	*S*. Choleraesuis @<br>4.6 × 109 Orally	Mild AEs	Significant increase in antibody and humoral response	100	[99]
		Mutant attenuated with *cya*, *crp* and *aroA* gene deletion	@3×10^9 CFU/animal orally with booster	*S*. Typhimurium<br><br>@ 10^6 CFU orally	No AEs	*Non- Significant increase in antibody response*	80	[100]
	Inactivated, Killed	Inactivated by Formalin and suspended into fenicated physiological serum	@ 10^8 CFU/animal Orally with booster	*S*. Typhimurium<br><br>@ 10^8 CFU orally	NR	*Non- Significant increase in antibody response*	87	[101]
		Inactivated by 0.8% formaldehyde without adjuvant	@ 2 × 10^9 CFU/ml/animal Intramuscularly (IM)	*S*. Choleraesuis<br><br>@5 × 10^8 CFU by oral gavage	No AEs	*Significant increase in antibody and humoral response*	100	[94]
		Crude cell lysate, inactivated with 37% formaldehyde (bacterin) with	@5 × 10^9 CFU/animal Orally with booster	*Salmonella* spp. @ 10^7 CFU Orally	No AEs	*Significant increase in antibody and humoral*	100	[102]

		Al $(OH)_3$ adjuvant				*response*		
		Inactivated with 1% formaldehyde with Al $(OH)_3$ adjuvant	@ 2 × 10^{10} CFU/animal Orally	*S*. Typhimurium @5 × 10^9 CFU Orally	Mild AEs	*Significant increase in antibody and humoral response*	100	[103]
Bovine	Killed, Inactivated	Whole bacterial cell lysate suspended in PBS	@2 ml/animal subcutaneously	S. Newport, S. Montevideo, S. Anatum @ 4.6 x10^7 CFU each SC	Mild AEs	*Significant increase in antibody and humoral response*	90	[104]
		Formalin-killed strain suspended in PBS	@2 ml/animal Intramuscularly	*S*. Typhimurium @ 1 x10^8 CFU, IM	No AEs	*Non- Significant increase in antibody response*	67	[105]
Caprine	Live attenuated	Rough attenuated	@10^9 CFU/ animal orally	*S*. Abortusovis @ 5 × 10^6 CFU, SC	Mild AEs	Significant increase in IgG and IgM response	90	[106]
		Mutant attenuated with *aroA gene* deletion	@ 1.6 × 10^9 CFU/animal Orally with booster SC	*S*. Abortusovis @ 1× 10^9 CFU, SC	No AEs	Significant increase in IgG and IgM response	100	[23]

References

1. Troxell, B., M. Mendoza, R. Ali, M. Koci, and H. Hassan, Attenuated Salmonella enterica serovar Typhimurium, strain NC983, is immunogenic, and protective against virulent Typhimurium challenges in mice. Vaccines, 2020. 8(4): p. 646.

2. Park, S., G. Won, and J.H. Lee, An attenuated Salmonella vaccine secreting Lawsonia intracellularis immunogenic antigens confers dual protection against porcine proliferative enteropathy and salmonellosis in a murine model. Journal of Veterinary Science, 2019. 20(3).

3. Milanez, G.P., C.H. Werle, M.R. Amorim, R.A. Ribeiro, L.H. Tibo, M.C. Roque-Barreira, A.F. Oliveira, and M. Brocchi, Hu-lacking mutants of Salmonella enterica enteritidis are highly attenuated and can induce protection in murine model of infection. Frontiers in microbiology, 2018. 9: p. 1780.

4. Fuche, F.J., J.A. Jones, G. Ramachandran, E.E. Higginson, R. Simon, and S.M. Tennant, Deletions in guaBA and htrA but not clpX or rfaL constitute a live-attenuated vaccine strain of Salmonella Newport to protect against serogroup C2-C3 Salmonella in mice. Human Vaccines

Immunotherapeutics, 2018.

5. Zhao, X., Q. Dai, R. Jia, D. Zhu, M. Liu, M. Wang, S. Chen, K. Sun, Q. Yang, and Y. Wu, Two novel Salmonella bivalent vaccines confer dual protection against two Salmonella serovars in mice. Frontiers in Cellular

Infection Microbiology, 2017. 7: p. 391.

6. Erova, T.E., M.L. Kirtley, E.C. Fitts, D. Ponnusamy, W.B. Baze, J.A. Andersson, Y. Cong, B.L. Tiner, J. Sha, and A.K. Chopra, Protective immunity elicited by oral immunization of mice with Salmonella enterica serovar Typhimurium Braun lipoprotein (Lpp) and acetyltransferase (MsbB) mutants. Frontiers in cellular infection microbiology, 2016. 6: p. 148.

7. Tennant, S.M., P. Schmidlein, R. Simon, M.F. Pasetti, J.E. Galen, and M.M. Levine, Refined live attenuated Salmonella enterica serovar Typhimurium and Enteritidis vaccines mediate homologous and heterologous serogroup protection in mice. Infection Immunity, 2015. 83(12): p. 4504-4512.

8. Matsui, H., Y. Isshiki, M. Eguchi, Y. Ogawa, and Y. Shimoji, Evaluation of the live vaccine efficacy of virulence plasmid-cured, and phoP-or aroA-deficient Salmonella enterica serovar Typhimurium in mice. Journal of Veterinary Medical Science, 2015. 77(2): p. 181-186.

9. Pati, N.B., V. Vishwakarma, S.K. Selvaraj, S. Dash, B. Saha, N. Singh, and M. Suar, Salmonella Typhimurium TTSS-2 deficient mig-14 mutant shows attenuation in immunocompromised mice and offers protection against wild-type Salmonella Typhimurium infection. BMC microbiology, 2013. 13: p. 1-10.

10. Xiong, K., Z. Chen, G. Xiang, J. Wang, X. Rao, F. Hu, and Y. Cong, Deletion of yncD gene in Salmonella enterica ssp. enterica serovar Typhi leads to attenuation in mouse model. FEMS Microbiology Letters, 2012. 328(1): p. 70-77.

11. Allam, U.S., M.G. Krishna, A. Lahiri, O. Joy, and D. Chakravortty, Salmonella enterica serovar Typhimurium lacking hfq gene confers protective immunity against murine typhoid. PLoS One, 2011. 6(2): p. e16667.

12. Santander, M.J. and R. Curtiss III, Salmonella enterica Serovars Typhi and Paratyphi A are avirulent in newborn and infant mice even when expressing virulence plasmid genes of Salmonella Typhimurium. Journal of infection in developing countries, 2010. 4(11): p. 723.

13. Pesciaroli, M., F. Aloisio, S. Ammendola, C. Pistoia, P. Petrucci, M. Tarantino, M. Francia, A. Battistoni, and P. Pasquali, An attenuated Salmonella enterica serovar Typhimurium strain lacking the ZnuABC transporter induces protection in a mouse intestinal model of Salmonella infection. Vaccine, 2011. 29(9): p. 1783-1790.

14. Hur, J., M.Y. Kim, and J.H. Lee, Evaluation of efficacy of a new live Salmonella Typhimurium vaccine candidate in a murine model. Comparative immunology, microbiology infectious diseases, 2011. 34(2): p. 171-177.

15. Piao, H.H., V.T.M. Tam, H.S. Na, H.J. Kim, P.Y. Ryu, S.Y. Kim, J.H. Rhee, H.E. Choy, S.W. Kim, and Y. Hong, Immunological responses induced by asd and wzy/asd mutant strains of Salmonella enterica serovar Typhimurium in BALB/c mice. The Journal of Microbiology, 2010. 48: p. 486-495.

16. Choi, J., D. Shin, and S. Ryu, Salmonella enterica serovar Typhimurium ruvB mutant can confer protection against salmonellosis in mice. Vaccine, 2010. 28(39): p. 6436-6444.

17. Ruan, P., X.-P. Xia, D. Sun, D.M. Ojcius, Y.-F. Mao, W.-Y. Yue, and J. Yan, Recombinant SpaO and H1a as immunogens for protection of mice from lethal infection with Salmonella paratyphi A: Implications for rational design of typhoid fever vaccines. Vaccine, 2008. 26(51): p. 6639-6644.

18. Liu, T., R. König, J. Sha, S.L. Agar, C.-T.K. Tseng, G.R. Klimpel, and A.K. Chopra, Immunological responses against Salmonella enterica serovar Typhimurium Braun lipoprotein and lipid A mutant strains in Swiss-Webster mice: potential use as live-attenuated vaccines. Microbial pathogenesis, 2008. 44(3): p. 224-237.

19. Lee, H.-Y., S.-A. Cho, I.-S. Lee, J.-H. Park, S.-H. Seok, M.-W. Baek, D.-J. Kim, S.-H. Lee, S.-J. Hur, and S.-J. Ban, Evaluation of phoP and rpoS mutants of Salmonella enterica serovar Typhi as attenuated typhoid vaccine candidates: virulence and protective immune responses in intranasally immunized mice. FEMS Immunology

Medical Microbiology, 2007. 51(2): p. 310-318.

20. Negi, V.D., S. Singhamahapatra, and D. Chakravortty, Salmonella enterica serovar Typhimurium strain lacking pmrG-HM-D provides excellent protection against salmonellosis in murine typhoid model. Vaccine, 2007. 25(29): p. 5315-5323.

21. Burns-Guydish, S.M., H. Zhao, D.K. Stevenson, and C.H. Contag, The Potential Salmonella aroA–Vaccine Strain Is Safe and Effective in Young BALB/c Mice. Neonatology, 2007. 91(2): p. 114-120.

22. Wu, C., L. Chen, and M.-L. Kuo, Attenuated Salmonella typhimurium reduces ovalbumin-induced airway inflammation and T-helper type 2 responses in mice. Clinical

Experimental Immunology, 2006. 145(1): p. 116-122.

23. Uzzau, S., G. Marogna, G.S. Leori, R. Curtiss III, G. Schianchi, B.A. Stocker, and S. Rubino, Virulence attenuation and live vaccine potential of aroA, crp cdt cya, and plasmid-cured mutants of Salmonella enterica serovar Abortusovis in mice and sheep. Infection

immunity, 2005. 73(7): p. 4302-4308.

24. Valentine, P.J., B.P. Devore, and F. Heffron, Identification of three highly attenuated Salmonella typhimurium mutants that are more immunogenic and protective in mice than a prototypical aroA mutant. Infection immunity, 1998. 66(7): p. 3378-3383.

25. Honda-Okubo, Y., R.T. Cartee, A. Thanawastien, J.S. Yang, K.P. Killeen, and N. Petrovsky, A typhoid fever protein capsular matrix vaccine candidate formulated with Advax-CpG adjuvant induces a robust and durable anti-typhoid Vi polysaccharide antibody response in mice, rabbits and nonhuman primates. Vaccine, 2022. 40(32): p. 4625-4634.

26. Maiti, S., D.R. Howlader, P. Halder, U. Bhaumik, M. Dutta, S. Dutta, and H. Koley, Bivalent non-typhoidal Salmonella outer membrane vesicles immunized mice sera confer passive protection against gastroenteritis in a suckling mice model. Vaccine, 2021. 39(2): p. 380-393.

27. Haque, S., S. Sengupta, D. Gupta, M.K. Bhan, R. Kumar, A. Khan, and B. Jailkhani, S. Typhi derived OmpC peptide conjugated with Vi-polysaccharide evokes better immune response than free Vi-polysaccharide in mice. Biologicals, 2019. 62: p. 50-56.

28. Baliban, S.M., B. Curtis, M.N. Amin, M.M. Levine, M.F. Pasetti, and R. Simon, Maternal antibodies elicited by immunization with an O-polysaccharide glycoconjugate vaccine protect infant mice against lethal Salmonella Typhimurium infection. Frontiers in immunology, 2019. 10: p. 2124.

29. Bajzert, J., M. Gorczykowski, and T. Stefaniak, Evaluation of the protective effect of immunization spf DBA/2J mice with selected bacterial, recombinant Hsp60 antigens during Salmonella Enteritidis challenge. Microbial pathogenesis, 2019. 128: p. 206-214.

30. Schuster, O., K.T. Sears, G. Ramachandran, F.J. Fuche, B. Curtis, S.M. Tennant, and R. Simon, Immunogenicity and protective efficacy against Salmonella C2-C3 infection in mice immunized with a glycoconjugate of S. Newport Core-O polysaccharide linked to the homologous serovar FliC protein. Human Vaccines Immunotherapeutics, 2018.

31. Liu, Q., J. Yi, K. Liang, X. Zhang, and Q. Liu, Outer membrane vesicles derived from Salmonella Enteritidis protect against the virulent wild-type strain infection in a mouse model. Journal of microbiology biotechnology, 2017. 27(8): p. 1519-1528.

32. Liu, Q., Q. Liu, X. Zhao, T. Liu, J. Yi, K. Liang, and Q. Kong, Immunogenicity and cross-protective efficacy induced by outer membrane proteins from Salmonella Typhimurium mutants with truncated LPS in mice. International journal of molecular sciences, 2016. 17(3): p. 416.

33. Yang, Y., C. Wan, H. Xu, Z.P. Aguilar, Q. Tan, F. Xu, W. Lai, Y. Xiong, and H. Wei, Identification of an outer membrane protein of Salmonella enterica serovar Typhimurium as a potential vaccine candidate for Salmonellosis in mice. Microbes Infection, 2013. 15(5): p. 388-398.

34. Hale, C., F. Bowe, D. Pickard, S. Clare, J.-F. Haeuw, U. Powers, N. Menager, P. Mastroeni, and G. Dougan, Evaluation of a novel Vi conjugate vaccine in a murine model of salmonellosis. Vaccine, 2006. 24(20): p. 4312-4320.

35. Ahmad, N., F. Deeba, S. Faisal, A. Khan, J. Agrewala, V. Dwivedi, and M. Owais, Role of fusogenic non-PC liposomes in elicitation of protective immune response against experimental murine salmonellosis. Biochimie, 2006. 88(10): p. 1391-1400.

36. Ochoa, J., J.M. Irache, I. Tamayo, A. Walz, V.G. DelVecchio, and C. Gamazo, Protective immunity of biodegradable nanoparticle-based vaccine against an experimental challenge with Salmonella Enteritidis in mice. Vaccine, 2007. 25(22): p. 4410-4419.

37. Román, B.S., V. Garrido, P.-M. Muñoz, L. Arribillaga, B. García, X. De Andrés, V. Zabaleta, C. Mansilla, I. Farrán, and I. Lasa, The extradomain a of fibronectin enhances the efficacy of lipopolysaccharide defective Salmonella bacterins as vaccines in mice. Veterinary research, 2012. 43(1): p. 1-11.

38. Cronly-Dillon, S., The relative potencies of heat-killed and acetone-killed vaccines against Salmonella typhimurium in mice. Epidemiology

Infection, 1972. 70(4): p. 597-603.

39. Moon, J., S. Kim, W. Kim, Z. Rao, J. Park, B. Park, and J. Hur, Protective efficacy of the recombinant lysozyme-PMAP36 fusion protein-inactivated Salmonella Typhimurium vaccine candidate via oral immunization in a murine model. Canadian Journal of Veterinary Research, 2020. 84(3): p. 241-244.

40. Jiao, H., H. Yang, D. Zhao, L. He, J. Chen, and G. Li, The enhanced immune responses induced by Salmonella enteritidis ghosts loaded with Neisseria gonorrhoeae porB against Salmonella in mice. FEMS Microbiology Letters, 2016. 363(22): p. fnw239.

41. Moon, J.Y., S.Y. Kim, W.K. Kim, Z. Rao, J.H. Park, J.Y. Mun, B. Kim, H.S. Choi, and J. Hur, Protective efficacy of a Salmonella Typhimurium ghost vaccine candidate constructed with a recombinant lysozyme-PMAP36 fusion protein in a murine model. Canadian Journal of Veterinary Research, 2017. 81(4): p. 297-303.

42. Kim, B., G. Won, and J.H. Lee, Construction of an inactivated typhoid vaccine candidate expressing Escherichia coli heat-labile enterotoxin B subunit and evaluation of its immunogenicity in a murine model. Journal of Medical Microbiology, 2017. 66(8): p. 1235-1243.

43. Hu, M., W. Zhao, H. Li, J. Gu, Q. Yan, X. Zhou, Z. Pan, G. Cui, and X. Jiao, Immunization with recombinant Salmonella expressing SspH2-EscI protects mice against wild type Salmonella infection. BMC veterinary research, 2018. 14: p. 1-8.

44. Ochoa-Repáraz, J., B. García, C. Solano, I. Lasa, J.M. Irache, and C. Gamazo, Protective ability of subcellular extracts from Salmonella Enteritidis and from a rough isogenic mutant against salmonellosis in mice. Vaccine, 2005. 23(12): p. 1491-1501.

45. Li, J., J. Qiu, Z. Huang, T. Liu, J. Pan, Q. Zhang, and Q.J.I.J.o.M.M. Liu, Reverse vaccinology approach for the identifications of potential vaccine candidates against Salmonella. 2021. 311(5): p. 151508.

46. Esmailnia, E., J. Amani, and S.L.M. Gargari, Identification of novel vaccine candidate against Salmonella enterica serovar Typhi by reverse vaccinology method and evaluation of its immunization. Genomics, 2020. 112(5): p. 3374-3381.

47. Sivasankar, C., C. Hewawaduge, and J.H.J.D. Lee, Screening of lipid-A related genes and development of low-endotoxicity live-attenuated Salmonella gallinarum by arnT deletion that elicits immune responses and protection against fowl typhoid in chickens. Developmental

Comparative immunology, microbiology, 2023. 145: p. 104707.

48. Jia, S., A.R. McWhorter, S. Khan, D.M. Andrews, G.J. Underwood, and K.K. Chousalkar, Investigation of a gel-based delivery method for the administration of a live, attenuated Salmonella Typhimurium vaccine. Veterinary Microbiology, 2023. 280: p. 109721.

49. Wang, X., X. Kang, M. Pan, M. Wang, J. Zhang, and H. Song, Evaluation of the protective immune response induced by an rfbG-deficient Salmonella enterica serovar enteritidis strain as a live attenuated DIVA (differentiation of infected and vaccinated animals) vaccine in chickens. Microbiology Spectrum, 2022. 10(6): p. e01574-22.

50. Shin, H., T.-M. La, H.-J. Lee, T. Kim, S.-u. Song, E. Park, G.-H. Park, I.-S. Choi, S.-Y. Park, and J.-B. Lee, Evaluation of Immune Responses and Protective Efficacy of a Novel Live Attenuated Salmonella Enteritidis Vaccine Candidate in Chickens. Vaccines, 2022. 10(9): p. 1405.

51. Akter, T., M. Nooruzzaman, S.M.S.H. Belal, M. Ahammed, A.J. Uddin, R. Parvin, M.A.H.N.A. Khan, M.A. Islam, and M.M. Hossain, Fowl typhoid live lyophilized vaccine applied at 3-month intervals protected layer chickens from Salmonella gallinarum infection and prevented cloacal shedding. Journal of Advanced Veterinary

Animal Research, 2022. 9(2): p. 301.

52. Senevirathne, A., C. Hewawaduge, and J.H. Lee, Assessing an O-antigen deficient, live attenuated Salmonella Gallinarium strain that is DIVA compatible, environmentally safe, and protects chickens against fowl typhoid. Developmental

Comparative Immunology, 2022. 133: p. 104433.

53. Yin, J., W. Xiong, X. Yuan, S. Li, L. Zhi, P. Pan, W. Sun, T. Yu, Q. He, and Z. Cheng, Salmonella Pullorum lacking srfA is attenuated, immunogenic and protective in chickens. Microbial Pathogenesis, 2021. 161: p. 105230.

54. Guo, Y., Y. Xu, X. Kang, D. Gu, Y. Jiao, C. Meng, P. Tang, X. Wang, C. Huang, and S. Geng, Immunogenic potential and protective efficacy of a sptP deletion mutant of Salmonella Enteritidis as a live vaccine for chickens against a lethal challenge. International Journal of Medical Microbiology, 2019. 309(8): p. 151337.

55. Ibe, M., D. Odimegwu, and E. Onuigbo, Alginate-coated chitosan microparticles encapsulating an oral plasmid-cured live Salmonella enterica serovar Gallinarum vaccine cause a higher expression of interferon-gamma in chickens compared to the parenteral live vaccine. Avian Pathology, 2019. 48(5): p. 423-428.

56. Muniz, E.C., R. Verdi, J.A. Leão, A. Back, and V.P.d. Nascimento, Evaluation of the effectiveness and safety of a genetically modified live vaccine in broilers challenged with Salmonella Heidelberg. Avian Pathology, 2017. 46(6): p. 676-682.

57. Guo, R., Y. Jiao, Z. Li, S. Zhu, X. Fei, S. Geng, Z. Pan, X. Chen, Q. Li, and X. Jiao, Safety, protective immunity, and DIVA capability of a rough mutant Salmonella Pullorum vaccine candidate in broilers. Frontiers in microbiology, 2017. 8: p. 547.

58. Lalsiamthara, J. and J.H. Lee, A live attenuated mutant of Salmonella Montevideo triggers IL-6, IFN-γ and IL-12 cytokines that co-related with humoral and cellular immune responses required for reduction of challenge bacterial load in experimental chickens. Comparative Immunology, Microbiology

Infectious Diseases, 2017. 50: p. 1-7.

59. Varmuzova, K., M. Faldynova, M. Elsheimer-Matulova, A. Sebkova, O. Polansky, H. Havlickova, F. Sisak, and I. Rychlik, Immune protection of chickens conferred by a vaccine consisting of attenuated strains of Salmonella Enteritidis, Typhimurium and Infantis. Veterinary research, 2016. 47(1): p. 1-11.

60. Penha Filho, R.A.C., S.J.A. Diaz, T. da Silva Medina, Y.-F. Chang, J.S. da Silva, and A. Berchieri Jr, Evaluation of protective immune response against fowl typhoid in chickens vaccinated with the attenuated strain Salmonella Gallinarum ΔcobSΔcbiA. Research in veterinary science, 2016. 107: p. 220-227.

61. Braukmann, M., P. Barrow, A. Berndt, and U.J.R.i.V.S. Methner, Combination of competitive exclusion and immunisation with a live Salmonella vaccine in newly hatched chickens: immunological and microbiological effects. Research in Veterinary Science, 2016. 107: p. 34-41.

62. Lalsiamthara, J., N.M. Kamble, and J.H. Lee, A live attenuated Salmonella Enteritidis secreting detoxified heat labile toxin enhances mucosal immunity and confers protection against wild-type challenge in chickens. Veterinary research, 2016. 47: p. 1-10.

63. De Cort, W., S. Geeraerts, V. Balan, M. Elroy, F. Haesebrouck, R. Ducatelle, and F. Van Immerseel, A Salmonella Enteritidis hilAssrAfliG deletion mutant is a safe live vaccine strain that confers protection against colonization by Salmonella Enteritidis in broilers. Vaccine, 2013. 31(44): p. 5104-5110.

64. Łaniewski, P., A. Mitra, K. Karaca, A. Khan, R. Prasad, R. Curtiss III, and K.L. Roland, Evaluation of protective efficacy of live attenuated Salmonella enterica serovar Gallinarum vaccine strains against fowl typhoid in chickens. Clinical

Vaccine Immunology, 2014. 21(9): p. 1267-1276.

65. Methner, U., P.A. Barrow, A. Berndt, and I. Rychlik, Salmonella Enteritidis with double deletion in phoP fliC—A potential live Salmonella vaccine candidate with novel characteristics for use in chickens. Vaccine, 2011. 29(17): p. 3248-3253.

66. Nandre, R.M. and J.H. Lee, Comparative evaluation of safety and efficacy of a live Salmonella gallinarum vaccine candidate secreting an adjuvant protein with SG9R in chickens. Veterinary Immunology

Immunopathology, 2014. 162(1-2): p. 51-58.

67. Matulova, M., H. Havlickova, F. Sisak, and I. Rychlik, Vaccination of chickens with Salmonella Pathogenicity Island (SPI) 1 and SPI2 defective mutants of Salmonella enterica serovar Enteritidis. Vaccine, 2012. 30(12): p. 2090-2097.

68. Elsheimer-Matulova, M., K. Varmuzova, K. Kyrova, H. Havlickova, F. Sisak, M. Rahman, and I. Rychlik, phoP, SPI1, SPI2 and aroA mutants of Salmonella Enteritidis induce a different immune response in chickens. Veterinary Research, 2015. 46: p. 1-12.

69. Copper, G.L., L.M. Venables, R.A. Nicholas, G.A. Cullen, and C.E. Hormaeche, Vaccination of chickens with chicken-derived Salmonella enteritidis phage type 4 aroA live oral Salmonella vaccines. Vaccine, 1992. 10(4): p. 247-254.

70. Acevedo-Villanueva, K., G. Akerele, W. Al-Hakeem, D. Adams, R. Gourapura, and R. Selvaraj, Immunization of broiler chickens with a killed chitosan nanoparticle Salmonella vaccine decreases Salmonella enterica serovar enteritidis load. Frontiers in Physiology, 2022. 13: p. 920777.

71. Clifton-Hadley, F., M. Breslin, L. Venables, K. Sprigings, S. Cooles, S. Houghton, and M.J. Woodward, A laboratory study of an inactivated bivalent iron restricted Salmonella enterica serovars Enteritidis and Typhimurium dual vaccine against Typhimurium challenge in chickens. Veterinary microbiology, 2002. 89(2-3): p. 167-179.

72. Crouch, C.F., C. Pugh, A. Patel, H. Brink, C. Wharmby, A. Watts, M.C. van Hulten, and S.P. de Vries, Reduction in intestinal colonization and invasion of internal organs after challenge by homologous and heterologous serovars of Salmonella enterica following vaccination of chickens with a novel trivalent inactivated Salmonella vaccine. Avian Pathology, 2020. 49(6): p. 666-677.

73. Ishida, Y., E. Sakai, K. Sato, E. Sugiyama, K. Mima, A. Taneno, H. Shimomura, L. Cui, and Y. Hirai, Induction of Mucosal Humoral Immunity by Subcutaneous Injection of an Oil-emulsion Vaccine against Salmonella enterica subsp. enterica serovar Enteritidis in Chickens. Food Safety, 2018. 6(4): p. 151-155.

74. Berghaus, R.D., S. Thayer, J. Maurer, and C. Hofacre, Effect of vaccinating breeder chickens with a killed Salmonella vaccine on Salmonella prevalences and loads in breeder and broiler chicken flocks. Journal of food protection, 2011(5): p. 727-734.

75. Gast, R.K., H.D. Stone, P.S. Holt, and C. Beard, Evaluation of the efficacy of an oil-emulsion bacterin for protecting chickens against Salmonella enteritidis. Avian Diseases, 1992: p. 992-999.

76. Suphabphant, W., M. York, and B. Pomeroy, Use of two vaccines (live G30D or killed RW16) in the prevention of Salmonella typhimurium infections in chickens. Avian Diseases, 1983: p. 602-615.

77. Maiti, S., P. Halder, S. Banerjee, M. Dutta, A.K. Mukhopadhyay, S. Dutta, and H. Koley, Development of a novel trivalent invasive non-typhoidal Salmonella outer membrane vesicles based vaccine against salmonellosis and fowl typhoid in chickens. Immunobiology, 2022. 227(2): p. 152183.

78. Chen, Y., K. Jie, B. Li, H. Yu, H. Ruan, J. Wu, X. Huang, and Q. Liu, Immunization with outer membrane vesicles derived from major outer membrane protein-deficient Salmonella Typhimurium mutants for cross protection against Salmonella enteritidis and avian pathogenic Escherichia coli O78 infection in chickens. Frontiers in Microbiology, 2020. 11: p. 588952.

79. Han, Y., S. Renu, V. Patil, J. Schrock, N. Feliciano-Ruiz, R. Selvaraj, and G.J. Renukaradhya, Mannose-modified chitosan-nanoparticle-based Salmonella subunit oralvaccine-induced immune response and efficacy in a challenge trial in broilers. Vaccines, 2020. 8(2): p. 299.

80. Renu, S., Y. Han, S. Dhakal, Y.S. Lakshmanappa, S. Ghimire, N. Feliciano-Ruiz, S. Senapati, B. Narasimhan, R. Selvaraj, and G. Renukaradhya, Chitosan-adjuvanted Salmonella subunit nanoparticle vaccine for poultry delivered through drinking water and feed. Carbohydrate polymers, 2020. 243: p. 116434.

81. Agarwal, R.K., K. Porteen, Z.B. Dubal, K. Asha, S. Shweta, and B. Ripan, Evaluation of recombinant outer membrane protein based vaccine against Salmonella Typhimurium in birds. Biologicals, 2013. 41(3): p. 162-168.

82. Okamura, M., W. Matsumoto, F. Seike, Y. Tanaka, C. Teratani, M. Tozuka, T. Kashimoto, K. Takehara, M. Nakamura, and Y. Yoshikawa, Efficacy of soluble recombinant FliC protein from Salmonella enterica serovar Enteritidis as a potential vaccine candidate against homologous challenge in chickens. Avian diseases, 2012. 56(2): p. 354-358.

83. Wisner, A.L., T.S. Desin, P.-K.S. Lam, E. Berberov, C.S. Mickael, H.G. Townsend, A.A. Potter, and W. Köster, Immunization of chickens with Salmonella enterica subspecies enterica serovar Enteritidis pathogenicity island-2 proteins. Veterinary microbiology, 2011. 153(3-4): p. 274-284.

84. Kaneshige, T., K. Yaguchi, and T. Ohgitani, Siderophore receptor IroN is an important protective antigen against Salmonella infection in chickens. Avian diseases, 2009. 53(4): p. 563-567.

85. Senevirathne, A., C. Hewawaduge, and J.H. Lee, Immunization of chickens with Salmonella Gallinarium ghosts expressing Salmonella Enteritidis NFliC-FimAC and CD40LC fusion antigen enhances cell-mediated immune responses and protects against wild-type challenges with both species. Developmental

Comparative immunology, microbiology, 2022. 126: p. 104265.

86. Won, G., A. Senevirathne, and J.H. Lee, Salmonella Enteritidis ghost vaccine carrying the hemagglutinin globular head (HA1) domain from H1N1 virus protects against salmonellosis and influenza in chickens. Vaccine, 2020. 38(28): p. 4387-4394.

87. Guo, R., S. Geng, H. Jiao, Z. Pan, X. Chen, and X. Jiao, Evaluation of protective efficacy of a novel inactivated Salmonella Pullorum ghost vaccine against virulent challenge in chickens. Veterinary Immunology

Immunopathology, 2016. 173: p. 27-33.

88. Jawale, C.V. and J.H. Lee, Characterization of a Salmonella Typhimurium ghost carrying an adjuvant protein as a vaccine candidate for the protection of chickens against virulent challenge. Avian Pathology, 2014. 43(6): p. 506-513.

89. Chaudhari, A.A., C.V. Jawale, S.W. Kim, and J.H. Lee, Construction of a Salmonella Gallinarum ghost as a novel inactivated vaccine candidate and its protective efficacy against fowl typhoid in chickens. Veterinary research, 2012. 43: p. 1-11.

90. Jiang, Z., X. Kang, Y. Song, X. Zhou, and M. Yue, Identification and Evaluation of Novel Antigen Candidates against Salmonella Pullorum Infection Using Reverse Vaccinology. Vaccines, 2023. 11(4): p. 865.

91. Peeters, L., J. Dewulf, F. Boyen, C. Brossé, T. Vandersmissen, G. Rasschaert, M. Heyndrickx, M. Cargnel, W. Mattheus, and F. Pasmans, Evaluation of group vaccination of sows and gilts against Salmonella Typhimurium with an attenuated vaccine in subclinically infected pig herds. Preventive Veterinary Medicine, 2020. 182: p. 104884.

92. Gil, C., C. Latasa, E. García-Ona, I. Lázaro, J. Labairu, M. Echeverz, S. Burgui, B. García, I. Lasa, and C. Solano, A DIVA vaccine strain lacking RpoS and the secondary messenger c-di-GMP for protection against salmonellosis in pigs. Veterinary research, 2020. 51: p. 1-10.

93. Theuß, T., E. Ueberham, J. Lehmann, T. Lindner, and S. Springer, Immunogenic potential of a Salmonella Typhimurium live vaccine for pigs against monophasic Salmonella Typhimurium DT 193. BMC veterinary research, 2017. 13(1): p. 1-8.

94. Alborali, G.L., J. Ruggeri, M. Pesciaroli, N. Martinelli, B. Chirullo, S. Ammendola, A. Battistoni, M.C. Ossiprandi, A. Corradi, and P. Pasquali, Prime-boost vaccination with attenuated Salmonella Typhimurium ΔznuABC and inactivated Salmonella Choleraesuis is protective against Salmonella Choleraesuis challenge infection in piglets. BMC veterinary research, 2017. 13: p. 1-9.

95. Foster, N., L. Richards, J. Higgins, T. Kanellos, and P. Barrow, Oral vaccination with a rough attenuated mutant of S. Infantis increases post-wean weight gain and prevents clinical signs of salmonellosis in S. Typhimurium challenged pigs. Research in Veterinary Science, 2016. 104: p. 152-159.

96. De Ridder, L., D. Maes, J. Dewulf, F. Pasmans, F. Boyen, F. Haesebrouck, E. Méroc, S. Roels, B. Leyman, and P. Butaye, Effect of a DIVA vaccine with and without in-feed use of coated calcium-butyrate on transmission of SalmonellaTyphimurium in pigs. BMC veterinary research, 2013. 9(1): p. 1-8.

97. Hur, J., S.O. Song, J.S. Lim, I.K. Chung, and J.H. Lee, Efficacy of a novel virulence gene-deleted Salmonella Typhimurium vaccine for protection against Salmonella infections in growing piglets. Veterinary Immunology

Immunopathology, 2011. 139(2-4): p. 250-256.

98. Domínguez-Bernal, G., A. Tierrez, A. Bartolomé, S. Martínez-Pulgarín, F.J. Salguero, J.A. Orden, and R. de la Fuente, Salmonella enterica serovar Choleraesuis derivatives harbouring deletions in rpoS and phoP regulatory genes are attenuated in pigs, and survive and multiply in porcine intestinal macrophages and fibroblasts, respectively. Veterinary Microbiology, 2008. 130(3-4): p. 298-311.

99. Chu, C.-Y., S.-Y. Wang, Z.-W. Chen, M.-S. Chien, J.-P. Huang, J.-J. Chen, L.-S. Hong, A.-L. Shiau, J.-L. Tsai, and C.-L. Wu, Heterologous protection in pigs induced by a plasmid-cured and crp gene-deleted Salmonella choleraesuis live vaccine. Vaccine, 2007. 25(41): p. 7031-7040.

100. Barrow, P., K. Page, and M. Lovell, The virulence for gnotobiotic pigs of live attenuated vaccine strains of Salmonella enterica serovars Typhimurium and Enteritidis. Vaccine, 2001. 19(25-26): p. 3432-3436.

101. Moura, E.A.G.d.O., D.G.d. Silva, C.H. Turco, T.V.C. Sanches, G.Y. Storino, H.M.d.S. Almeida, M.L. Mechler-Dreibi, I.P. Rabelo, K. Sonalio, and L.G.d. Oliveira, Salmonella Bacterin Vaccination Decreases Shedding and Colonization of Salmonella Typhimurium in Pigs. Microorganisms, 2021. 9(6): p. 1163.

102. Arguello, H., A. Carvajal, G. Naharro, and P. Rubio, Evaluation of protection conferred by a Salmonella Typhimurium inactivated vaccine in Salmonella-infected finishing pig farms. Comparative immunology, microbiology

infectious diseases, 2013. 36(5): p. 489-498.

103. Hur, J., J.H. Lee, Y.H. Choi, and M.S. Choi, Efficacy of two virulence gene-deleted Salmonella Typhimurium vaccine candidate for Salmonella infections in weaning piglets. Journal of the Preventive Veterinary Medicine 2013. 37(4): p. 177-180.

104. Dodd, C.C., D.G. Renter, D.U. Thomson, and T. Nagaraja, Evaluation of the effects of a commercially available Salmonella Newport siderophore receptor and porin protein vaccine on fecal shedding of Salmonella bacteria and health and performance of feedlot cattle. American journal of veterinary research, 2011. 72(2): p. 239-247.

105. Robertsson, J. and H. Carlsson, ELISA for measurement of antibody response to a killed Salmonella typhimurium vaccine in cattle. Zentralblatt für Veterinärmedizin Reihe B, 1980. 27(1): p. 28-35.

106. García-Seco, T., C. Montbrau, M. Fontseca, R. March, M. Sitja, L. Domínguez, and J. Bezos, Efficacy of a Salmonella enterica serovar Abortusovis (S. Abortusovis) inactivated vaccine in experimentally infected gestating ewes. Research in Veterinary Science, 2021. 135: p. 486-494.
